# Phage-Functionalized Magnetic Separation for Rapid and Selective Detection of *Escherichia coli*

**DOI:** 10.1021/acsomega.6c02406

**Published:** 2026-06-15

**Authors:** Marco Eigenfeld, Benjamin Schneider, Dagmar Kolb, Ana Lisac, Ales Podgornik, Sebastian P. Schwaminger

**Affiliations:** † NanoLab, Division of Medicinal Chemistry, Otto Loewi Research Center, 31475 Medical University of Graz, Neue Stiftingtalstraße 6, 8010 Graz, Austria; ‡ BioTechMed-Graz, Mozartgasse 12/II, 8010 Graz, Austria; § Core Facility Ultrastructural Analysis, Medical University of Graz, Neue Stiftingtalstraße 6, 8010 Graz, Austria; ∥ Division of Cell Biology, Histology and Embryology,, Gottfried Schatz Research Center, Medical University of Graz, Neue Stiftingtalstraße 6, 8010 Graz, Austria; ⊥ Faculty of Chemistry and Chemical Technology, 37663 University of Ljubljana, Večna pot, 113, Ljubljana 1000, Slovenia

## Abstract

Selective bacterial preconcentration remains a key challenge for improving downstream molecular detection. Here, we report carboxymethyl-dextran (CMD) coated magnetic nanoparticle clusters functionalized with lytic T4 bacteriophages (phage@CMD) for selective capture of *Escherichia coli*. Iron-oxide particles were synthesized by coprecipitation and characterized by dynamic light scattering (DLS), Fourier transform infrared (FT-IR) spectroscopy, and electron microscopy, revealing CMD-stabilized nanoparticle clusters with a hydrodynamic diameter of approximately 181 nm with moderate dispersity (PDI = 0.267). T4 phages were immobilized via EDC/Sulfo-NHS chemistry, yielding a functional loading of ∼5.54 × 10^12^ PFU-equivalent g^–1^ (∼0.053 phage per particle). An empirical Langmuir-type analysis was used solely to estimate surface loading capacity. Phage-functionalized particles enabled magnetic capture of *E. coli*, resulting in experimentally determined separation efficiencies of ∼20–40%, verified by CFU depletion and PCR analysis of particle-associated fractions. Magnetic preconcentration improved the practical PCR input threshold compared to direct amplification of untreated suspensions. While the study represents a proof-of-concept in laboratory media, limitations including random phage orientation, partial loss of activity after immobilization, and lack of complex-matrix validation are discussed. These findings support the use of phage@CMD as a modular magnetic preconcentration platform for bacteria prior to nucleic-acid–based analysis.

## Graphical abstract

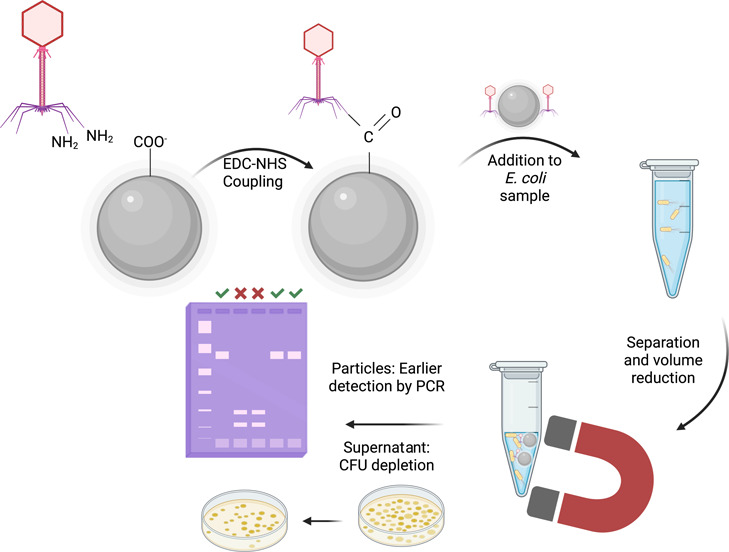

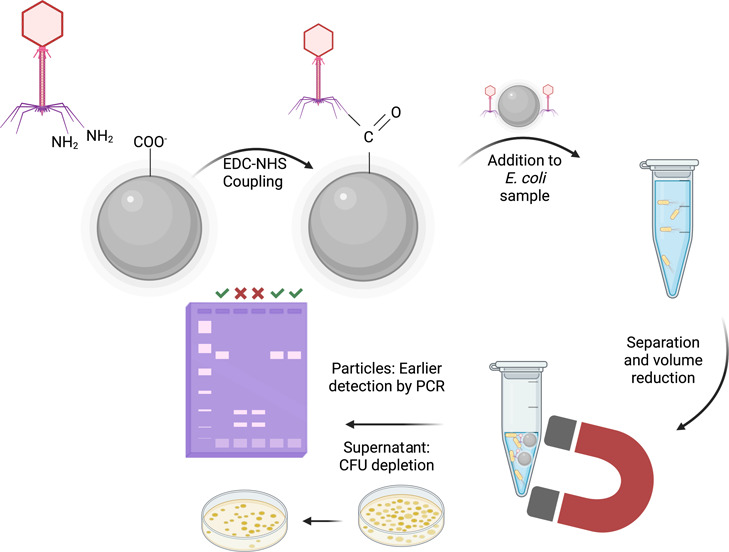

## Introduction

Reliable and selective detection of bacterial contaminants or indicator organisms is vital across sectors such as clinical diagnostics, food safety, and environmental monitoring. Conventional detection methods, including culture-based techniques and molecular assays such as PCR or ELISA, often require extended processing times and laboratory infrastructure, limiting their applicability for routine or high-throughput screening workflows.^1,2^ These limitations have motivated the development of alternative bioanalytical strategies that combine selective target recognition with efficient sample preconcentration.^3^ In the context of this study, phages are used primarily as selective capture ligands rather than as viability reporters.

Among emerging recognition elements, bacteriophages have attracted considerable interest due to their inherent host specificity, environmental robustness, and compatibility with diverse sensing and separation platforms.^2,4–7^ Phages, viruses that infect bacteria, selectively infect metabolically active bacterial hosts, although adsorption to nonviable cells may still occur when bacterial surface receptors remain intact.^7,8^ This makes phages attractive biological recognition elements for bacterial capture and enrichment applications. In particular, lytic phages provide strong and selective host interactions that can be exploited for targeted separation prior to downstream analytical readout.^7,9,10^

To translate these biological properties into functional materials, stable immobilization of phages onto solid supports is required. Covalent attachment via carbodiimide chemistry, typically employing EDC (1-ethyl-3-(3-(dimethylamino)­propyl)­carbodiimide) in combination with NHS or Sulfo-NHS (*N*-hydroxysulfosuccinimide), is widely used to couple amine-containing phage proteins to carboxyl-functionalized surfaces.^11,12^ While this approach provides robust attachment and scalability, it generally results in nonsite-specific immobilization and may lead to heterogeneous phage orientation and partial loss of biological activity.^6,12^ Nevertheless, such coupling strategies remain attractive due to their simplicity and compatibility with diverse materials.

Magnetic iron oxide nanoparticles represent particularly useful supports for bioseparation because they enable efficient target enrichment through external magnetic fields,^2,13,14^ and have been widely used in pathogen detection systems including surface-enhanced Raman scattering (SERS)-based magnetic nanoprobes.^15,16^ Depending on synthesis conditions, these systems frequently form multicore magnetic nanoparticle clusters rather than isolated single-core nanoparticles, which exhibit strong field-induced magnetization and efficient magnetic responsiveness. Surface modification with carboxymethyl dextran (CMD) enhances colloidal stability and provides reactive carboxyl groups for covalent functionalization, making CMD-coated magnetic particles well suited for biomolecule immobilization.^11,13,14^

Previous studies have demonstrated phage-functionalized magnetic particles for bacterial capture and detection, including T4-conjugated systems.^2,6,17–21^ However, the relationship between immobilization strategy, phage loading, and functional capture efficiency remains insufficiently quantified. In many cases, immobilization efficiency is reported without directly linking surface loading to biological performance, making it difficult to evaluate practical limitations such as orientation effects or activity loss after coupling.^6,12^

While magnetic nanoparticle-based pathogen separation has been extensively explored, including early work on SERS-enabled magnetic nanoprobes,^22^ and phage-functionalized magnetic systems such as T4-conjugated particles,^21^ these studies primarily focus on detection platform development or parameter-dependent capture performance, rather than linking immobilization chemistry to functional capture efficiency. More recent approaches using phage-derived binding proteins^23^ emphasize integration into point-of-care assay platforms (e.g., magnetic separation coupled with paper-based colorimetric sensors and smartphone readout), while treating immobilization chemistry as a fixed step rather than linking surface functionalization to capture efficiency.

The aim of this study is not to introduce a new detection platform, but to provide a mechanistic assessment of factors governing phage-mediated magnetic separation, enabling identification of key limitations relevant for future system optimization.

In this work, we investigate the covalent immobilization of lytic T4 bacteriophages onto CMD-coated magnetic nanoparticle clusters using EDC/Sulfo-NHS chemistry and evaluate their functional performance for selective *Escherichia coli* (*E. coli*) capture. Rather than introducing a new coupling chemistry, the focus lies on assessing phage loading, capture efficiency, and the relationship between immobilization and biological function. The resulting phage-functionalized particles are investigated as a magnetic preconcentration platform enabling downstream PCR-based analysis. Particular emphasis is placed on linking immobilization-derived phage loading to experimentally observed capture efficiency. This allows identification of practical limitations associated with nonoriented covalent coupling.

## Material and Methods

### Bacteriophage and Bacterial Strains

A lytic *Myoviridae* bacteriophage T4 (DSM 4505) and its host strain *E. coli* K-12 MG1655 (DSM 18039) were used in all experiments.

High-titer phage lysate was prepared as follows: *E. coli* cultures were grown in Lysogeny Broth (LB, pH 7) and incubated overnight at 37 °C. The following day, a fresh culture was inoculated and allowed to grow at 37 °C until the exponential phase began (∼1.5 h after inoculation). To maximize phage production, infection was initiated at this time point.

Phage T4 (2.3 × 10^10^ PFU mL^–1^) was added at a multiplicity of infection (MOI) of 0.1. After 5 h of incubation to allow complete lysis, the lysate was treated with 1% chloroform and centrifuged to remove bacterial debris. The aqueous supernatant containing the high-titer phage preparation was ultrafiltered using Amicon centrifugal filters (30 kDa MWCO). The filtrate was adjusted to the original volume with SM buffer (5.8 g NaCl, 50 mL 1 M Tris–HCl pH 7.5, 2.0 g MgSO_4_·7H_2_O, 5 mL 2% gelatin), and this step was repeated once. The final preparation was used for subsequent experiments.

For specificity assessment, *Lactobacillus plantarum* (strain ATCC 8014) was cultured in MRS broth at 37 °C overnight, as mentioned by.^24^

### Phage Quantification

A phage stock with a defined concentration (2.3 × 10^10^ PFU mL^–1^, determined by DLA) was used to calibrate the BCA assay. Based on this reference, a correlation between BCA signal and PFU was established for this specific preparation. This calibration was subsequently used to estimate phage concentrations in derived samples.

Phage titers (PFU mL^–1^) were determined by the double-agar overlay plaque assay. Briefly, 50 μL of the phage sample was mixed with 450 μL of SM buffer and serially diluted (Table S1).

For the overlay, 5 mL of LB medium containing 0.7% (w/v) agar was mixed with 100 μL of an overnight *E. coli* culture and poured onto LB agar plates (1.2% agar) to form a bacterial lawn. Five microliters of each dilution (in triplicate) were spotted, plates were incubated at 37 °C, and plaques were counted after 12 h (exemplary shown in Figure S12).

Phage concentration was also quantified using the BCA assay (Pierce BCA Protein Assay Kit, A65453, ThermoFisher). A range of phage dilutions corresponding to concentrations between 2 × 10^8^ and 2.3 × 10^10^ PFU/mL was prepared in SM buffer. Ten μL of each dilution were mixed with 200 μL BCA working reagent, incubated at 37 °C for 30 min, and measured at 561 nm in a 96-well plate.

### Infection Kinetics Assay

To determine the MOI at which phage infection is considered complete—defined here as the condition in which nearly all susceptible host cells have been infected during the initial adsorption phase—one-step growth experiments were conducted. These experiments also provided preliminary insights into burst timing, which refers to the interval between phage adsorption and host cell lysis, during which intracellular phage replication and assembly occur. *E. coli* DSM 18039 cultures were grown overnight in LB medium at 37 °C with shaking at 160 rpm. The following day, 1 mL of culture was inoculated into 100 mL of fresh LB and incubated until an OD_600_ of 0.5–0.6 was reached. In a 24-well microtiter plate, 280 μL of LB, 280 μL of phage suspension (prepared at various concentrations with SM buffer), and 560 μL of *E. coli* were combined, resulting in MOIs ranging from 10.7 to 0.0001. The plate was incubated at 37 °C, and OD_600_ readings were recorded over a 12 h period to assess infection kinetics.

Negative control is represented by *E. coli* culture incubated with LB buffer instead of phage suspension. An additional control is given by substitution of phage suspension with SM buffer.

### Lysis Time Determination

A one-step growth experiment was performed to determine the burst size and lysis time of bacteriophage T4. *E. coli* DSM 18039 cultures were grown overnight in LB broth at 37 °C with shaking at 160 rpm for 16 h. Subsequently, 1 mL of the overnight culture was inoculated into 9 mL of fresh LB and incubated under the same conditions until the culture reached an OD_600_ of approximately 5.3.

Phages were added to the *E. coli* cultures to achieve MOIs of approximately 0.02 and 0.002, calculated based on a bacterial concentration of 8 × 10^8^ CFU mL^–1^ at OD_600_ = 5.3. Specifically, 7.48 μL of 10-fold and 100-fold diluted phage suspensions (stock: 2.3 × 10^10^ PFU mL^–1^) were added to 1 mL of bacterial suspension. The mixtures were incubated at room temperature for 5 min to allow phage adsorption. Unbound phages were then removed by centrifugation at 10,000*g* for 5 min at room temperature, the supernatants were discarded, and the pellets were resuspended in 1 mL of fresh LB broth.

The cultures were then incubated at 37 °C with agitation at 250 rpm using a thermoshaker.

Samples were collected at regular time points (5, 10, 15, 20, 25, 30, 40, 50, 60, 70, 80, 90, and 100 min postinfection). Each sample was immediately plated for PFU enumeration on DLA using 5 μL droplets at three serial dilutions (10^–4^, 10^–5^, and 10^–6^).

All experiments were performed in triplicate, and results are presented as mean ± standard deviation.

### Binding Kinetics

*E. coli* DSM 18039 was grown overnight in LB broth at 37 °C with shaking (300 rpm), diluted 1:100 into fresh LB, and incubated to mid log phase (OD_600_ = 0.5). Adsorption assays were performed in LB supplemented with 10 mM MgSO_4_ and 10 mM CaCl_2_ at 37 °C. At t_0_, 900 μL of the bacterial suspension was mixed with 100 μL of phage diluted to 4 × 10^7^ PFU/mL, yielding a final concentration of 4 × 10^8^ cells/mL and 4 × 10^6^ PFU/mL (MOI = 0.01). At defined intervals (15, 30, 45, 60, 75, 90, 105, 120, 150, 180 s), 100 μL samples were withdrawn and immediately diluted 1:10 and 1:100 into ice-cold LB lacking divalent cations (SM-ΔMg) to halt further adsorption. Free phage titers were determined by the double-layer agar method by plating 5 μL of this 1:100 dilution, as well as a further 10-fold dilution (1:1000). The adsorption rate constant *k* was calculated from the slope of $\ln\left[\frac{P\left(t\right)}{P_{0}}\right]$ versus time, using $\frac{dP}{dt}=-kBP$ , where *P* is the free phage concentration and *B* the bacterial density.

### Synthesis of CMD Particles

Synthesis was conducted according to Turrina et al.^25^ Briefly a solution of 347 mg FeCl_2_·4H_2_O (1.75 mmol) and 567 mg FeCl_3_ (3.5 mmol) in 20 mL degassed deionized water was prepared. Twenty mL of CMD solution (10 g/L; i.e., 0.2 g CMD) and 2.5 mL of 25% ammonium hydroxide were mixed thoroughly.

The CMD-ammonia solution was heated to 85 °C and stirred at 100 rpm. The iron salt solution was then added slowly under vigorous stirring. Upon addition, the mixture immediately turned black, indicating nanoparticle formation. Stirring was continued for 1 h.

After synthesis, the suspension was transferred to a Duran bottle for washing. Magnetic decantation was performed using an external NdFeB magnet. The supernatant was discarded, and the pellet was washed with degassed deionized water (2–3 times) and ethanol (2 times). This was repeated until the conductivity dropped below 200 μS/cm or the pH reached ∼7. The pH was monitored using a calibrated pH meter.

To determine nanoparticle concentration, three 1.5 mL Eppendorf tubes were dried at 60 °C and weighed. Each tube was filled with 1 mL of the well-mixed sonicated particle suspension and dried overnight. The resulting dry masses were determined gravimetrically. The average dry mass was 16 mg/mL. The particle suspension was stored in closed containers at 4 °C.

Based on the coprecipitation synthesis route and hydrodynamic size distribution, the resulting structures are referred to as magnetic nanoparticle clusters (MNCs) throughout this work.

### Immobilization onto CMD Particles

CMD-coated nanoparticles were functionalized following the Merck “Microsphere Coupling” protocol with minor modifications. Particles were washed and activated for subsequent ligand coupling. Initially, 625 μL of a 16 mg/mL solution (10% (w/v)) nanoparticle suspension was transferred to a low-protein binding microcentrifuge tube. The particles were washed three times with 1 mL of activation buffer consisting of 10 mM MES (2-(*N*-morpholino) ethanesulfonic acid), pH 6.0. Between each washing step, the particles were separated from the supernatant using centrifugation (10 min, 14.000*g*). The supernatant was carefully decanted, and the pellet was thoroughly resuspended by repeated pipetting.

Immediately before activation, the coupling reagents were prepared fresh. A 200 mM EDC solution was prepared by dissolving 19.2 mg EDC in 500 μL of Milli-Q water, while a 200 mM Sulfo-NHS solution was prepared by dissolving 21.7 mg Sulfo-NHS in 500 μL of MES buffer. To 1 mL of the washed nanoparticle suspension, 24 μL of the freshly prepared EDC solution and 240 μL of the Sulfo-NHS solution were added rapidly. The mixture was vortexed and incubated on a shaker for 30 min at room temperature (600 rpm) to allow activation of carboxyl groups on the particle surface.

Following activation, the nanoparticles were separated by centrifugation, and the supernatant was removed. The particles were washed three times with 1 mL of activation buffer, with centrifugal separation and resuspension performed between each wash. After the final wash, the particles were resuspended in 1000 μL of activation buffer and sonicated to ensure a homogeneous, monodisperse suspension suitable for subsequent ligand coupling. Fourier transform infrared (FT-IR) spectroscopy was determined at each step to support successful coupling.

EDC/Sulfo-NHS activation of CMD generates NHS esters that react with primary amines (e.g., lysine side chains) on the phage to form stable amide bonds.^12^ High-resolution structural and engineering studies on bacteriophage T4 show that the capsid head provides a large, solvent-exposed protein surface, and that this head region (via Hoc/Soc decoration proteins and capsid proteins) is widely used as the primary scaffold for dense chemical and genetic modification.^26^ In addition, several biosensor implementations and reviews describe head-down, tail-up immobilization strategies, exploiting the surface properties and charge of the head to anchor the phage while preserving tail accessibility for host recognition.^27^ Phage orientation was not experimentally determined in this study.

Following activation, the CMD-coated nanoparticle suspension was resuspended in 700 μL of activation buffer. Two coupling approaches were tested (1 and 10 μL particles in 990 μL phage suspension).

To assess binding efficiency, the particles were coupled at 12 concentrations, ranging from the dilutions of the stock (2.2 × 10^14^) highest point (1.02 × 10^10^ PFU mL^–1^) down to 1% of that stock (1.02 × 10^8^ PFU mL^–1^).

Coupling proceeded overnight at RT with gentle shaking. After magnetic separation, unbound phages in the supernatant were quantified by BCA assay. The Langmuir-type model was applied as an empirical fitting approach to estimate apparent surface loading capacity. Since EDC/Sulfo-NHS coupling is irreversible, the model is not interpreted as an equilibrium binding process.

To block remaining active sites and minimize nonspecific binding, the nanoparticles were resuspended in 1 mL of blocking buffer (50 mM Tris, pH 8.0, 0.5% (w/v) BSA), resuspended, and mixed on a shaker overnight at room temperature. After magnetic separation, the supernatant was removed, and the particles were washed again with 1 mL of fresh blocking buffer. Resuspension was achieved through pipetting. The final separation step was carried out using a magnet to pellet the particles before proceeding with *E. coli* further characterization of the CMD@phage particles.

### Electron Microscopy (TEM/SEM)

Transmission electron microscopy (TEM) and scanning electron microscopy (SEM) were used to evaluate nanoparticle morphology, cluster structure, and particle–bacteria interactions after functionalization and separation experiments.

For TEM analysis, diluted nanoparticle suspensions were applied onto glow-discharged, carbon-coated copper grids and incubated for 1 min to allow particle adsorption. Excess suspension was carefully removed using filter paper strips. Negative staining was performed by applying a drop of 1% (w/v) aqueous uranyl acetate solution onto the grid for 1 min. The stain was subsequently blotted off with filter paper, and the grids were air-dried at room temperature.

Specimens were examined using a FEI Tecnai G2 (FEI, Eindhoven, Netherlands) equipped with a Gatan Rio 16 high-speed CMOS camera. Imaging was performed at an acceleration voltage of 120 kV. TEM was used to assess particle size distribution, multicore cluster architecture, and structural integrity of the CMD-coated magnetic nanoparticle clusters.

For scanning electron microscopy, bacteria–particle suspensions were deposited onto glass coverslips and fixed using 2% (w/v) paraformaldehyde in 0.1 M phosphate-buffered saline (PBS, pH 7.4) and 2.5% glutaraldehyde in 0.1 M PBS (pH 7.4). Samples were postfixed with 1% osmium tetroxide (Electron Microscopy Sciences) for 1 h at room temperature to enhance membrane contrast and structural preservation.

Following fixation, specimens were dehydrated stepwise in a graded ethanol series (30–96% and 100%, v/v). Hexamethyldisilazane (HMDS; Merck | Sigma-Aldrich) was applied as a chemical drying agent to minimize structural collapse during drying. The coverslips were then mounted onto aluminum stubs using conductive double-sided carbon tape and sputter-coated with a thin conductive metal layer prior to imaging.

SEM imaging was performed using a Sigma 500VP FE-SEM (Zeiss, Oberkochen, Germany) equipped with a secondary electron detector and operated at an acceleration voltage of 5 kV. SEM was used to visualize particle morphology and interactions between phage-functionalized particles and bacterial cells.

### Vibrating Sample Magnetmetry (VSM)

Magnetic properties of the CMD-coated magnetic nanoparticle clusters were characterized using vibrating sample magnetometry (VSM). Prior to measurement, particle suspensions were freeze-dried overnight to obtain dry powder samples. The dried material was collected, weighed, and used for magnetization analysis.

Magnetization measurements were performed using a Lake Shore vibrating sample magnetometer at room temperature under an applied magnetic field range of ±3.2 T. Magnetization values were normalized to the dry sample mass to allow comparison of magnetic response between samples. The measurements were used to evaluate the magnetic behavior and field-induced magnetization of the CMD-coated nanoparticle clusters relevant for magnetic separation applications.

### FT-IR

Nanoparticle functionalization was verified by ATR-FTIR (Spectrum Two, PerkinElmer).

A 1.5 μL aliquot from each modification step was deposited on the ATR crystal and dried under cold air. Spectra were recorded from 4000–400 cm^–1^ (4 scans, DTGS detector), background-corrected, and normalized.

### Dynamic Light Scattering (DLS)

Hydrodynamic diameter of CMD coated IONs was measured by DLS (Cordouan Vasco) using a 0.1 g L^–1^ particle dispersion in Milli-Q water (pH 6.8).

DLS was performed on two types of suspensions: (i) free T4 phages, and (ii) IONs@phage conjugates. Measurements were carried out in activation buffer at pH 6. For the phage-only samples, a refractive index (RI) of 1.45 was used (typical for proteinaceous particles). For the IONs@phage conjugates, we assumed a higher effective refractive index (RI = 2.3), reflecting the dominant contribution of iron oxide over the attached phage proteins. The higher RI accounts for the composite nature of the conjugates and was used consistently for all ION-containing samples.

### Microorganism Separation Using phage@CMD System

*E. coli* DSM 18039 cultures were diluted to low cell densities. Based on our calibration, an OD_600_ of 0.0005 corresponds to approximately 7.5 × 10^4^ CFU/mL. In total, 49 μL of this suspension (≈3.7 × 10^3^ cells) was incubated for 5 min with 1 μL of phage-functionalized CMD particles (≈143 μg, assuming no particle loss). After incubation, the particles were collected by centrifugation (3000*g*; 1 min) for reproducible recovery of small volumes, washed once with PBS, and resuspended in 5 μL. From this, 1 μL was used as template for PCR. As a control, 0.5 μL of phage-functionalized CMD particles without bacteria was included in the PCR.

To assess the influence of particle dosage on capture performance, additional experiments were performed using increased volumes of phage-functionalized particles (2 μL instead of 1 μL) under otherwise identical conditions. Capture performance was evaluated by plating the supernatant and quantifying remaining colony-forming units (CFU). PCR analysis was not performed for these experiments.

To determine the remaining cell concentration of *E. coli* after separation, the supernatant from the incubation was plated on LB agar. For this, 1 μL of supernatant was mixed with 4 μL PBS and streaked onto plates and incubated overnight at 37 °C for CFU enumeration.

### PCR Detection Limit

To determine the minimum number of *E. coli* cells detectable by PCR, cultures with OD_600_ ranging from 0.0001 to 0.1 were prepared. The OD_600_ value of 0.1 was adjusted photometrically and validated by flow cytometry to confirm the corresponding cell concentration of 1,500,000 cells in 10 μL.

For PCR, 1 μL of each cell suspension was mixed with 10.5 μL nuclease-free water, 0.5 μL of each primer, and 12.5 μL OneTaq 2× Master Mix, yielding a final reaction volume of 25 μL. The primers used were 27F (10 μM, 5′-AGAGTTTGATCMTGGCTCAG-3′) and 1492R (10 μM, 5′-TACCTTGTTACGACTT-3′). PCR cycling conditions included an annealing step at 38 °C and an elongation step of 2 min.

PCR products were mixed with 6× loading dye, and 6 μL of each sample was loaded onto a 1% agarose gel containing Midori Green.

For specificity assessment, *L. plantarum* was diluted spanning OD_600_ values of 0.1–0.001 were prepared to refine the minimum detectable cell concentration. For each dilution, 1 μL of culture was used as PCR template following the same reaction composition described below. PCR products were analyzed on 1% agarose gels to determine the lowest OD_600_ yielding a visible band.

### Statistical Analysis

In phage infection studies, the Poisson distribution is used to model how many phages infect each bacterium.

The formula for the probability of a bacterium being infected by exactly *k* phages is

$$P\left(k\right)=\frac{e^{-\lambda }\cdot \lambda^{k}}{k!}$$

where: λ is the MOI and *e* ≈ 2.71828.

The specific growth rate (μ) was calculated using the natural logarithm of optical density (OD_600_) over time, assuming exponential growth. For a given time interval [*t*_1_,*t*_2_], μ was estimated by linear regression according to the equation

$$\mu =\frac{d\ln\left(OD\right)}{dt}$$

Practically, this corresponds to the slope of the best-fit line of ln­(OD_i_) versus time *t*_*i*_ within the selected interval. Only OD values greater than zero were included. The regression was performed in R using the ln­() function.

## Results and Discussion

### Phage Quantification

Phage concentration determined by double-layer agar (DLA) ranged from (2.67 ± 1.15) × 10^9^ to (5.53 ± 0.64) × 10^9^ PFU/mL, while BCA-based estimation yielded (1.07 ± 0.17) × 10^10^ PFU-equivalent mL^–1^ after calibration. It should be noted that this approach assumes a constant ratio between total protein content and infectious phage particles and is therefore specific to the analyzed stock. The BCA assay does not quantify infective units but total protein content. The ∼2–5-fold discrepancy aligns with prior observations that biochemical assays may overestimate noninfectious particles or under-represent aggregated virions.^28,29^ Conversely, DLA quantifies only infective units yet may be influenced by plaque variability or “lysis from without”.^30,31^ Given the operational simplicity and rapidity of BCA, we used it for stock quantification in downstream experiments while acknowledging its limitations.

Taken together, these observations highlight the need for complementary quantification approaches. While DLA remains the gold standard for measuring infective phages, biochemical and physical methods like BCA offer valuable insights into total particle load and sample integrity, particularly where high-throughput or rapid quantification is required. For further experiments, BCA measurements were expressed as PFU-equivalent values based on calibration with a reference stock.

### Infection Kinetic

Monitoring OD_600_ over 12 h at varying MOIs revealed distinct infection dynamics. At MOIs below approximately 1, *E. coli* growth was sustained, suggesting that bacterial replication either outpaced or balanced phage-induced lysis (Figure 1). In contrast, at higher MOIs (≥1), OD_600_ values declined rapidly, indicating that phage infection outpaced bacterial proliferation and led to efficient host lysis.

**Figure 1:**
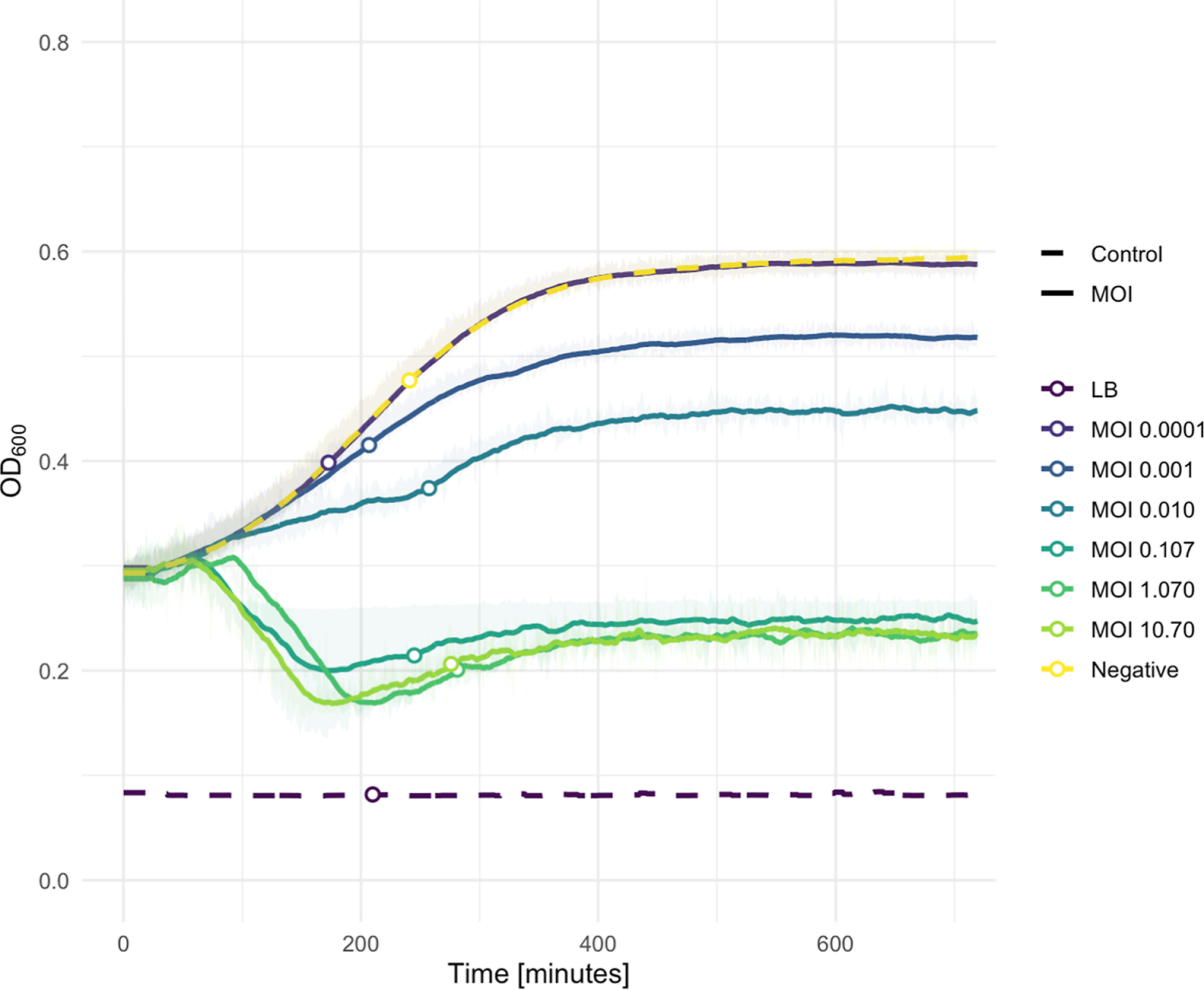
Infection kinetics of *E. coli* at different MOIs were compared to two controls: (1) a negative control consisting of *E. coli* cultured in LB broth without phages (phage volume replaced with media), and (2) a blank control containing only LB broth without *E. coli* inoculation. Each condition was tested in triplicate (*N* = 3).

To better interpret infection efficiency at different MOIs, we considered the expected distribution of phage adsorption events based on the Poisson model. The Poisson distribution describes the probability that a given bacterium is infected by 0, 1, or multiple phages, depending on the MOI. This statistical model assumes random, independent adsorption events and is widely used to describe early infection dynamics in phage-bacteria systems. For example, at an MOI of 1, approximately 63% of bacteria are expected to be infected (*P* ≥ 1), while at an MOI of 10, nearly all bacteria (>99.9%) are infected, most by multiple phages. These probabilities align with our observed infection kinetics: higher MOIs resulted in rapid and widespread lysis, whereas lower MOIs allowed partial bacterial growth due to a significant proportion of uninfected cells.

*E. coli* cells were in exponential growth phase throughout the experiment, during which T4 phage infection proceeds efficiently. At MOI 10, a marked decline in OD_600_ occurred within the first 100 min, suggesting that most cells were synchronously infected and underwent lysis within a single replication cycle. Phage infection follows a classical lytic cycle involving adsorption to the bacterial surface, DNA injection, replication of phage components, assembly of progeny, and host cell lysis to release new virions. The efficiency of this process is highly dependent on the phage’s host range and the susceptibility of the target bacteria.^32^ Under the tested conditions, the drop in OD_600_ suggests that the full lytic cycle—including latent period and lysis—was completed within 100 min. This time point was therefore used as an approximation of lysis time, defined here as the interval between adsorption and cell lysis.

The primary objective of this experiment was to compare lysis efficiency across MOIs and examine how infection timing correlates with bacterial density changes. This also enabled an estimation of phage lysis timing under high-infection conditions.

The limited impact on *E. coli* growth at MOIs below 0.1, along with moderate suppression up to an MOI of 1, is consistent with findings by Geng et al. (2024), who showed that lysis timing and infection efficiency depend heavily on MOI.^33^ They also noted that at very high MOIs, bacterial survival can paradoxically be preserved due to rapid lysis events or “lysis from without,” which may be less effective in reducing the total viable population. This trend is also reflected in our results, where even low MOIs significantly altered growth dynamics compared to phage-free controls.

Additional studies support that low MOIs (e.g., 0.1) generally lead to inefficient bacterial clearance, with final OD_600_ values often exceeding 0.5. In contrast, MOIs ≥1 more reliably resulted in OD_600_ values below 0.5, indicating more effective lysis and bacterial removal.^34^

### Binding Kinetics

Free-phage counts from the DLA assay (5 μL; 1:1000) were back-calculated to PFU mL^–1^ and fitted to the mass-action model *P*(*t*) = *P*_0_ *e* ^–*kBt*^ over the initial linear window (15–90 s) (Figures 2 and S3). Under the applied assay conditions (MOI = 0.01), semilog regression yielded a slope of -*kB* = −1.53 min^–1^ (95% CI), corresponding to an adsorption rate constant of *k* = 3.83 × 10^–9^ mL cell^–1^ min^–1^ (95% CI). The associated binding half-time was *t*_1/2_ = 27.2 s. From this fit, the times required to reach 90%, 95% and 99% adsorption of free phages were 90.3 s, 122.4 and 180.6 s, respectively (*R* ^2^ = 0.943). These results confirm a pseudo–first-order loss of free T4 during the first 1.5 min, with kinetic parameters consistent with reported T4 adsorption constants under ion-replete, well-mixed conditions.

**Figure 2:**
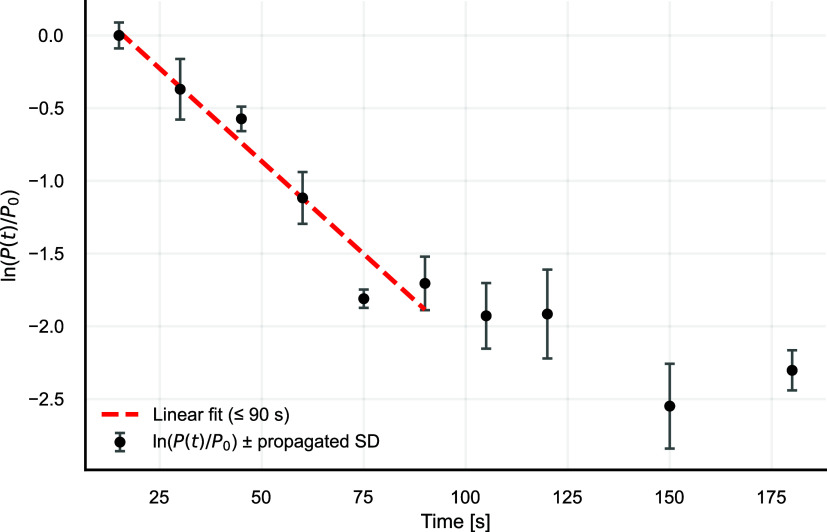
Time-dependent reduction of free phages (PFU/mL) during binding to *E. coli*, indicating phage adsorption kinetics.

### Lysis Time Determination

Lysis timing was assessed using a one-step growth assay at MOIs of 0.002 and 0.02, with phage concentration determined via BCA assay. Samples were collected at defined intervals (5 to 100 min postinfection) and plated at three dilutions (10^–4^, 10^–5^, and 10^–6^) using a double-layer agar method.

Up to approximately 40 min postinfection, PFUs were detectable only at the 10^–4^ dilution and remained relatively stable. This plateau reflects the presence of infective centers—bacteria that have adsorbed phages and are undergoing intracellular replication—but not yet released progeny phages. The number of free, plaque-forming virions thus remained low during this latent phase. Around 40–50 min, a sharp increase in PFU counts was observed, and higher dilutions became countable, indicating the onset of lysis and release of progeny virions.

This rise in PFU concentration marks the end of the latent period—the time between adsorption and initial lysis. PFU levels continued to increase and reached a plateau by approximately 80 min, suggesting that the full lytic cycle (lysis time) was completed by this time under the given conditions (Figures 3 and S1). These results align with previous reports of T4 phage dynamics, which show latent periods of 20–45 min and complete burst cycles within 80 min, depending on environmental and host factors.^35^

**Figure 3:**
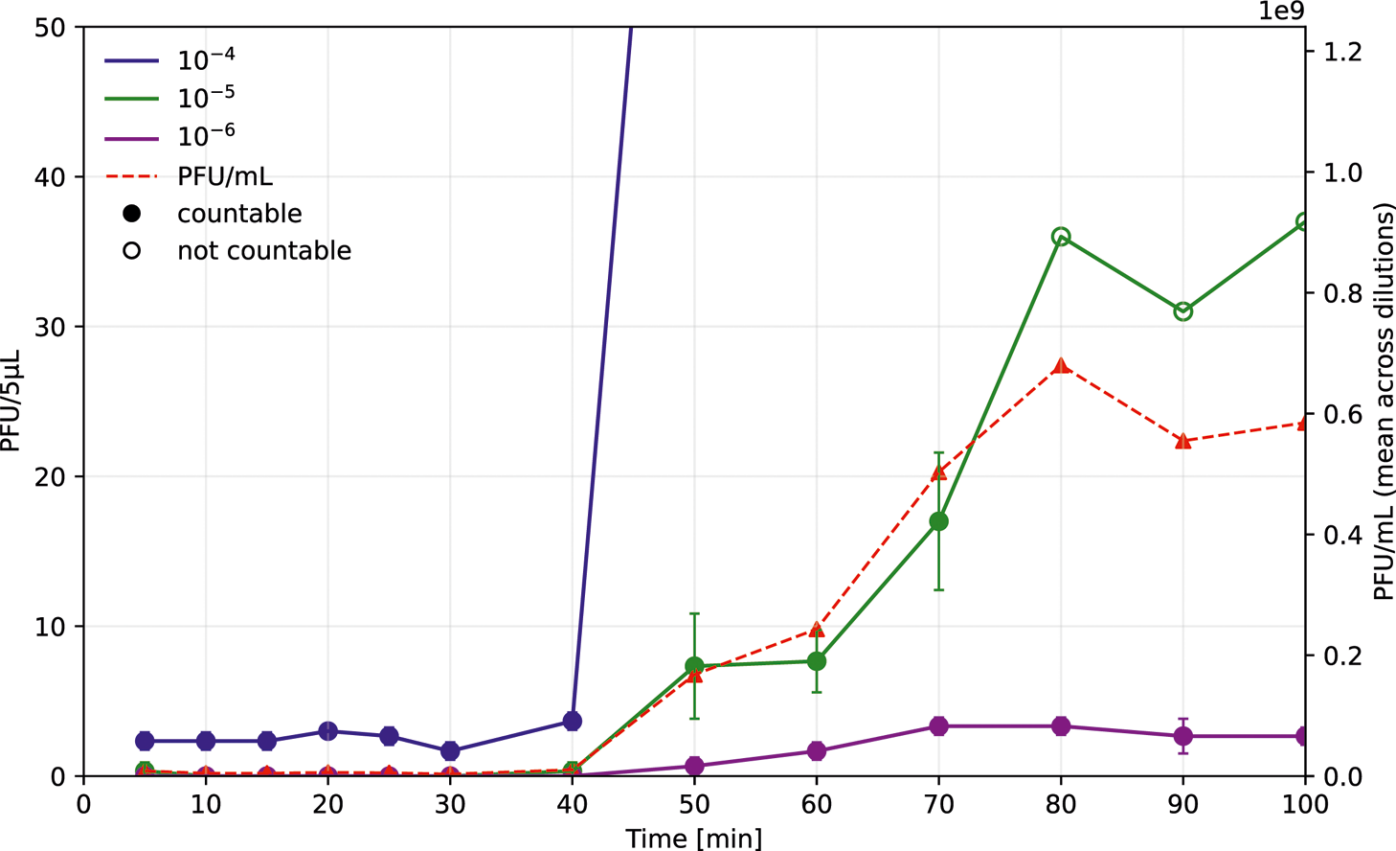
Time-course of bacteriophage release during a one-step growth experiment at MOI 0.02. Samples were taken at indicated time points postinfection and plated as 5 μL droplets on double-layer agar (DLA) at three serial dilutions (10^–4^, 10^–5^, and 10^–6^).

The slight decline in PFU numbers after 80 min may reflect secondary adsorption, where released phages reinfect remaining susceptible host cells, leading to an apparent reduction in free, plaque-forming units.^35^ Alternatively, this plateau and decrease may also relate to the molecular packaging behavior of T4. Vafabakhsh et al. demonstrated that the T4 packaging motor operates in dynamic bursts, alternating between active and paused states, and is dependent on rapid ATP turnover to overcome increasing internal capsid pressure.^36^ These dynamics could contribute to variability in packaging rates and delayed or staggered virion release, particularly in asynchronous infections at low MOI.

When normalized to the number of input phages, both MOIs achieved comparable overall phage amplification, indicating a consistent burst size per infected cell (Figure S2). However, greater variability was observed at the lower MOI, likely due to stochastic variation in initial infection events and the smaller number of infected cells contributing to plaque counts. These variations can influence the accuracy of early stage measurements where plaque numbers are low and amplification is still minimal.

The kinetic parameters derived from this one-step growth assay—including latent period, lysis time, and phage yield—were subsequently used to inform time point selection for downstream biosensor assays. This ensured that sampling occurred during biologically relevant phases of the infection cycle, capturing active lysis and progeny release for reliable functional evaluation of immobilized phages.

### Physicochemical Characterization of the phage@CMD System

Transmission electron microscopy (TEM) was performed to verify the morphology of free T4 bacteriophages prior to immobilization (Figure S4). The images revealed the characteristic head–tail structure of T4 phages, consisting of an icosahedral capsid and a contractile tail, consistent with previously reported morphology. This analysis served as a structural reference for subsequent immobilization experiments and supported the integrity of the phage preparation used for particle functionalization.

TEM imaging of the CMD-coated nanoparticles revealed multicore magnetic nanoparticle clusters, consistent with the aggregation of primary iron-oxide nanocrystals during the coprecipitation process (Figure 4). The observed cluster morphology supports the formation of magnetically responsive nanoparticle assemblies suitable for subsequent surface functionalization.

**Figure 4:**
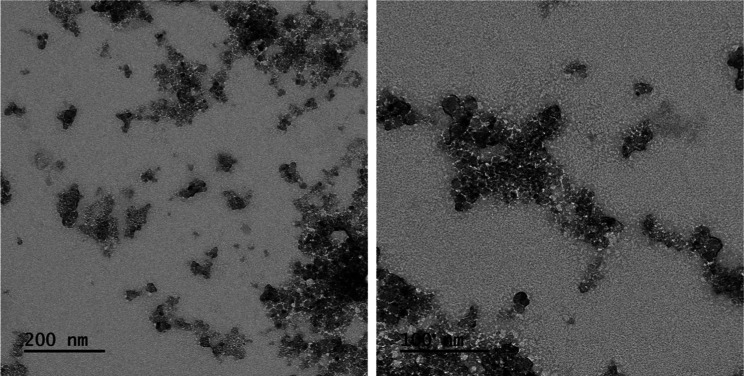
TEM images of CMD-coated magnetic nanoparticle clusters synthesized by coprecipitation.

The magnetic properties of the CMD-coated nanoparticle clusters were evaluated by vibrating sample magnetometry (Figure S5). The magnetization curve exhibits a characteristic sigmoidal shape with negligible hysteresis, indicating the absence of significant remanent magnetization and coercivity at room temperature. This behavior is consistent with superparamagnetic behavior. A saturation magnetization of approximately 40 emu g^–1^ was observed, confirming sufficient field-induced magnetization for magnetic separation. The absence of pronounced hysteresis suggests efficient magnetic collection under an external field while minimizing irreversible particle aggregation after field removal.

FT-IR spectroscopy confirmed the successful surface functionalization of the particles with CMD (Figure S6). Characteristic absorption bands were observed at approximately 1600 cm^–1^ (asymmetric COO^–^ stretch) and 1410–1450 cm^–1^ (symmetric COO^–^ stretch), verifying the presence of carboxymethyl groups. Additionally, strong bands in the 1000–1150 cm^–1^ region, corresponding to C–O–C vibrations, are consistent with the glycosidic linkages of the dextran backbone. Pyranose-related bands between 850 and 930 cm^–1^ were also observed.

DLS analysis revealed an intensity-weighted Z-average hydrodynamic diameter of 181 nm with a polydispersity index (PDI) of 0.267, indicating a moderately narrow size distribution (Figure S7). Number-weighted distributions showed smaller particle diameters (∼62 nm), consistent with the presence of multicore clusters observed by TEM.

### Immobilization

Adsorption data were fitted using a Langmuir-type model to estimate the apparent surface loading capacity of phages on CMD-coated particles (Figure 5). Nonlinear regression yielded an apparent maximum loading capacity of *B*_max_ = 7.76 × 10^12^ PFU g^–1^ and an apparent constant *K*_*d*_ = 1.20 × 10^9^ PFU mL^–1^. Since EDC/Sulfo-NHS coupling results in irreversible covalent bond formation, the Langmuir model was used here solely as an empirical fitting approach to estimate surface loading capacity rather than to describe equilibrium affinity.

**Figure 5:**
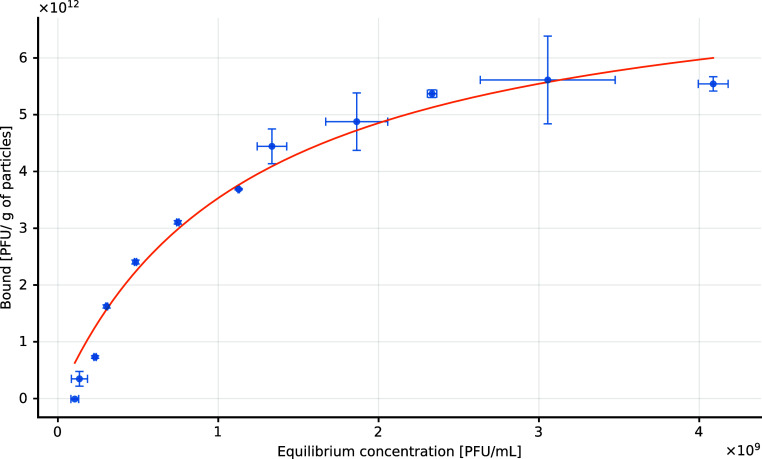
Adsorption isotherm of the binding of phage toward CMD particles using EDC/NHS.

In the immobilization assay, 100 μL of particle suspension (1 mg particles) was incubated with 900 μL of phage stock (1.07 × 10^10^ PFU mL^–1^), corresponding to a total phage input of 9.63 × 10^9^ PFU (9.63 × 10^12^ PFU g^–1^). This value is moderately higher than the experimentally determined maximum loading of 5.54 × 10^12^ PFU g^–1^, indicating that an excess of phages was present during immobilization, but not sufficient to ensure complete surface saturation under the applied conditions. The incomplete utilization of available binding sites likely reflects heterogeneous adsorption behavior, steric hindrance and clustering effects, as well as heterogeneous phage orientation during covalent attachment.^37,38^ Consequently, subsequent immobilization experiments were performed using undiluted or higher-concentration phage suspensions to approach maximal practical loading.

The phage loading per particle was estimated assuming spherical particles with a hydrodynamic radius of 70.7 ± 16.7 nm derived from DLS measurements. The number-based hydrodynamic diameter was used for particle number estimation, as it better reflects the dominant particle population compared to intensity-weighted values. Based on the calculated particle mass and particle number per gram, the experimentally determined loading corresponds to approximately 0.053 phages per particle (≈0.88 phages μm^–2^). Considering the size distribution obtained by DLS, this value represents an estimate and may vary depending on particle size. Assuming random distribution, this corresponds to ∼5% of particles carrying at least one phage (Poisson statistics), indicating a predominantly inactive particle population. However, it should be noted that this estimation is based on particle numbers derived from DLS measurements assuming discrete particles. In practice, the system consists of multicore nanoparticle clusters and aggregates, and the calculated values therefore represent an approximation rather than an exact description of the functional particle population.

Literature reports on phage surface densities, including values on planar substrates,^37,39^ provide useful reference points; however, direct comparison to the present system is limited. In contrast to well-defined planar surfaces, the multicore nanoparticle clusters used here exhibit curvature, aggregation, and heterogeneous surface accessibility, which influence both the effective availability of binding sites and the apparent surface density. Consequently, the calculated values should be interpreted within the context of clustered colloidal systems rather than as directly comparable to planar substrate data.

DLS measurements further supported phage attachment (Figure 6). Free T4 phages showed a D_50_ (number distribution) of 137.7 ± 1.1 nm, while CMD-coated particles exhibited a smaller D_50_ of 70.7 ± 16.7 nm. After phage conjugation, the hydrodynamic diameter increased to 133.6 ± 20.4 nm, consistent with the formation of particle–phage conjugates. The comparable apparent sizes of free phages and conjugates likely reflect differences in scattering behavior and deviations from the spherical particle assumption inherent to DLS analysis. Overall, the observed size shift supports successful phage immobilization and formation of stable phage@CMD conjugates. TEM confirms the presence of phages associated with the particle surface (Figure S8); however, due to the limited projection and local sampling, it does not provide information on phage orientation.

**Figure 6:**
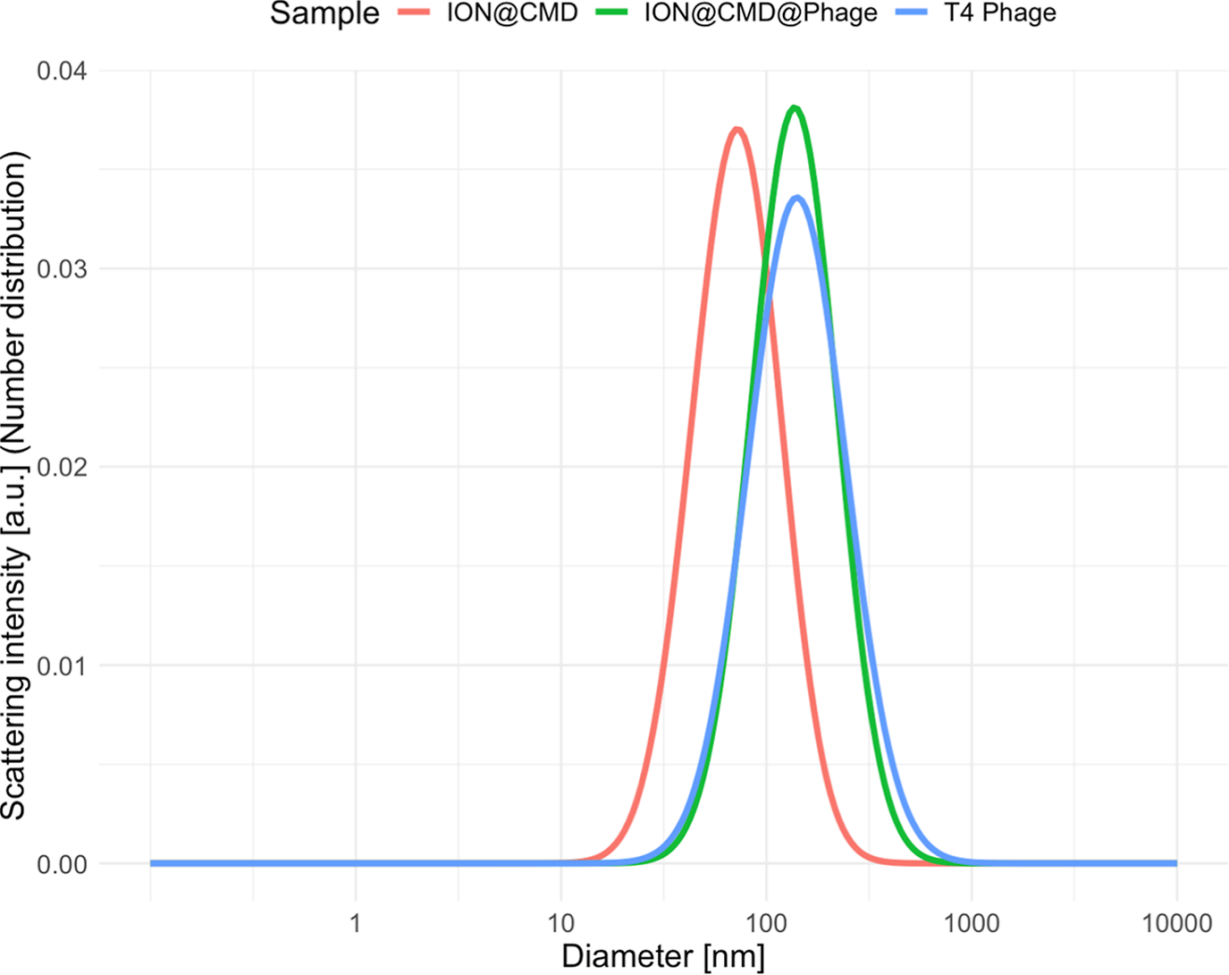
Number distribution of the samples T4 phage, ION@CMD and ION@Phages in comparison to free phages.

### FT-IR

FT-IR spectra were recorded at each step of the EDC/NHS coupling procedure. Figure S6 illustrates the spectrum of washed CMD particles in binding buffer (10 mM MES, pH 6), together with CMD particles after 30 min incubation with EDC/NHS followed by three washing steps (Figure S9). These spectra allow the assessment of chemical changes associated with activation of carboxyl groups.

Figure 7 shows the spectra of CMD particles after overnight incubation of the EDC/NHS-activated particles with phages, followed by three washing steps with binding buffer.

**Figure 7:**
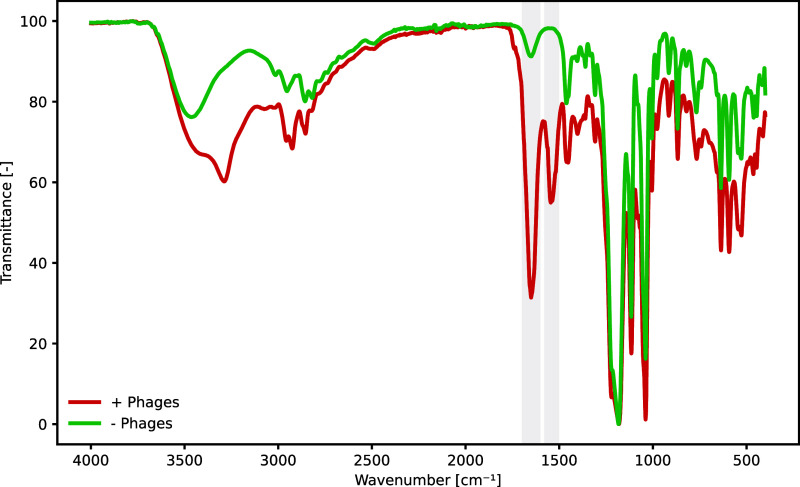
FT-IR spectra of CMD particles after EDC/NHS activation and incubation overnight with phages (+Phages, red) or without phages (–Phages, green). The gray-shaded regions highlight the Amide II band (1500–1580 cm^–1^) and the Amide I band (1600–1700 cm^–1^). Spectra were min–max normalized for visualization.

Figure 7 compares the FT-IR spectra of CMD particles with phages (+, red) and without phages (−, green). Distinct absorptions are observed in the Amide I (∼1650 cm^–1^) and Amide II (∼1540 cm^–1^) regions, corresponding to protein backbone vibrations.^40,41^ These features are consistent with the presence of phage coat proteins bound to the CMD surface.

The carbohydrate fingerprint region (1200–900 cm^–1^) shows characteristic saccharide bands of CMD, confirming retention of the polysaccharide backbone. The absence of notable changes in this region indicates that the CMD scaffold remains structurally intact following coupling.

Control experiments performed without EDC/Sulfo-NHS activation showed no detectable phage-associated signals after washing (Figure S10), indicating that nonspecific adsorption is negligible under the applied conditions. Together with the FT-IR results, this supports that phage attachment is primarily mediated by the applied coupling chemistry, although covalent bonding cannot be conclusively proven by the presented data alone.

### Effect of Magnetic Preconcentration on PCR Detectability

PCR detectability of *E. coli* was first evaluated without prior magnetic separation. For this purpose, bacterial cultures were diluted to defined optical densities, and 1 μL of each suspension was used directly as template for conventional end point PCR. Clear amplification signals were obtained down to OD_600_ = 0.001 (≈1.5 × 10^5^ CFU mL^–1^), whereas samples at OD_600_ = 0.0005 (≈7.5 × 10^4^ CFU mL^–1^) did not produce detectable bands on agarose gels (Figure 8). Control reactions containing only EDC/NHS-activated CMD particles yielded no amplification products, confirming that the particles themselves did not interfere with the PCR reaction.

**Figure 8:**
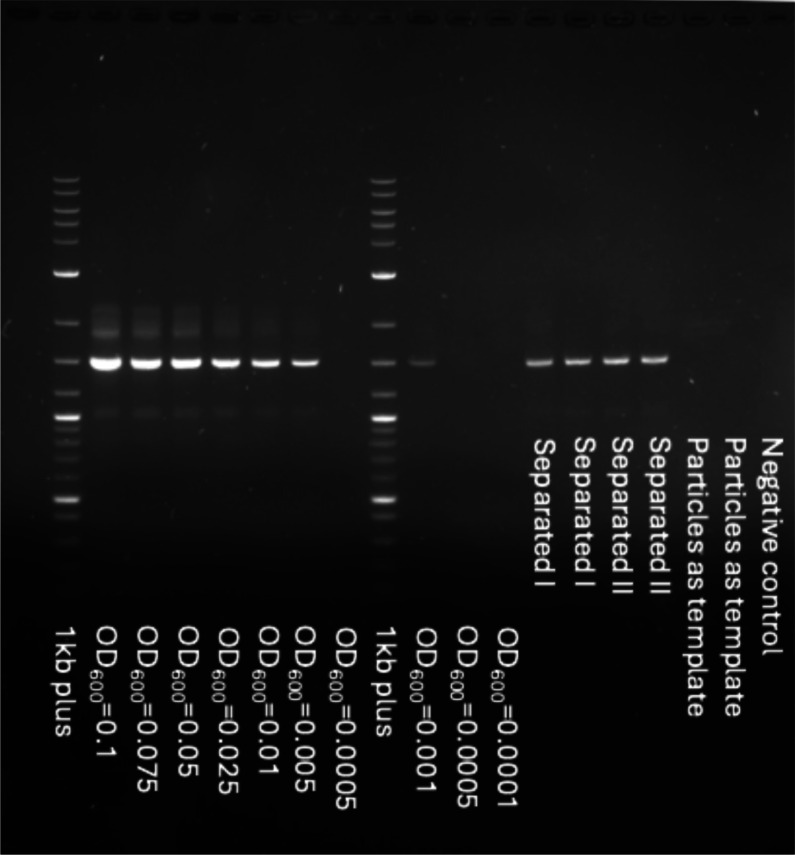
Agarose gel electrophoresis of PCR products obtained from *E. coli* cultures at various optical densities (OD_600_) and from the separated *E. coli* fraction after incubation with phage-functionalized CMD particles.

The observed PCR threshold is consistent with typical sensitivity ranges reported for conventional end point PCR, which generally requires higher template concentrations than quantitative PCR approaches. Literature reports describe detectable amplification in pure cultures typically in the range of 10^3^–10^4^ CFU mL^–1^ or higher depending on assay conditions, primer design, and template volume, supporting the experimental observations obtained here.^42–44^

Following magnetic separation with phage-functionalized CMD particles, PCR analysis of the particle-associated fractions yielded clear amplification bands for both tested particle concentrations (Separated I and Separated II). Notably, samples initially corresponding to OD_600_ = 0.0005 produced detectable PCR products after magnetic preconcentration, whereas untreated suspensions at the same concentration remained below the detection threshold. The intensity of the amplification bands was comparable to that obtained from untreated suspensions at OD_600_ ≈ 0.005, indicating that magnetic separation increased the amount of bacterial DNA entering the PCR reaction.

These results demonstrate that phage-mediated magnetic separation acts as an effective preconcentration step prior to molecular analysis. Rather than defining a formal analytical limit of detection, the experiments show that magnetic enrichment improves practical PCR detectability by increasing the number of captured cells available for amplification. The observed improvement is consistent with the capture efficiencies determined independently from CFU reduction experiments.

To further evaluate selectivity, *L. plantarum* was analyzed using the same universal 16S primers (27*F*/1492R). While initial cultures produced only weak diffuse background signals at high cell densities, no defined amplicon was observed in the particle-associated fraction (Figure S11). Because the primers are capable of amplifying both *E. coli* and *L. plantarum*, the absence of a distinct band indicates that *L. plantarum* cells were not enriched by the phage@CMD particles. These results confirm that enrichment specificity originates from phage–host interactions rather than primer selectivity. However, only a single nonhost organism was tested, and broader selectivity cannot be concluded from these data.

Overall, PCR analysis served primarily as a qualitative verification of bacterial enrichment on the particles, demonstrating that magnetic preconcentration with phage@CMD increases PCR detectability through selective bacterial capture.

### *E. coli* Separation with phage@CMD

The bacterial capture performance of phage@CMD particles was quantified by colony-forming unit (CFU) analysis before and after magnetic separation. Plating experiments revealed a decrease from 71 ± 7 CFU prior to separation to 43 ± 5 CFU in the supernatant after incubation with phage-functionalized particles. This corresponds to a capture efficiency of 39 ± 9%, demonstrating measurable depletion of viable *E. coli* cells from the liquid phase through phage-mediated magnetic separation.

An independent estimate derived from PCR-based enrichment experiments indicated a minimum capture efficiency of approximately 20%, based on the number of cells required to produce visible amplification bands. The agreement between CFU-based and PCR-based estimates confirms that phage@CMD particles consistently enrich bacterial cells within a moderate capture range of approximately 20–40%.

Increasing the particle volume from 1 to 2 μL reduced the number of detectable *E. coli* in the supernatant from 83 ± 4 CFU to 18 ± 5 CFU, corresponding to approximately 78 ± 6% removal based on CFU quantification. This increase reflects the higher number of available binding sites at elevated particle concentrations and does not indicate an improvement in intrinsic capture efficiency per particle.

Notably, the increase in removal is not strictly proportional within experimental uncertainty to the increase in particle volume, suggesting that simply increasing the number of particles does not fully compensate for limitations in functional accessibility. In the context of the low average loading (∼0.053 phage per particle), these results indicate that only a fraction of immobilized phages effectively contributes to bacterial capture. This behavior is consistent with steric hindrance, random phage orientation, and limited surface accessibility, particularly within multicore particle clusters where parts of the surface may be shielded from interaction with solution-phase bacteria.^45,46^

To evaluate long-term stability, phage-functionalized CMD particles were stored in blocking buffer at 4 °C for up to three months. Functional activity was assessed using a double-layer agar assay. No detectable reduction in phage activity was observed over the storage period, as lytic activity was retained up to a dilution of 10^–5^, indicating that covalent immobilization combined with storage in blocking buffer preserves functional phage activity over extended periods.

The observed capture efficiency is consistent with the immobilization analysis presented above and reflects the contribution of surface-bound phages to bacterial binding.

Control experiments (Figure S10, Supporting Information) demonstrate that phages do not adsorb to CMD-coated particles in the absence of EDC/Sulfo-NHS activation, confirming that immobilization is governed by covalent coupling rather than nonspecific interactions.

Accordingly, bacterial capture is attributed predominantly to phage-mediated interactions at the particle surface.

Although substantial phage loading was achieved, only a fraction of surface-bound phages is expected to remain functionally accessible due to nonoriented covalent coupling, steric hindrance, and heterogeneous surface presentation.^4^ Consequently, high immobilization density does not directly translate into proportional capture efficiency, and the experimentally observed values reflect practical limitations of nonsite-specific phage immobilization.

Selectivity of the separation process was assessed using *L. plantarum* as a nonhost control. No significant reduction of viable *L. plantarum* cells was observed in the supernatant after incubation with phage@CMD particles (CFU before: 24 ± 6; after: 20 ± 3). In agreement with this result, PCR analysis of the particle-associated fraction showed only faint diffuse background signals without a defined amplicon, indicating that no enrichment occurred. These findings are consistent with phage–host specificity under the tested conditions. The specificity arises from phage–receptor interactions on the bacterial surface.^47,48^ However, adsorption to nonviable cells with intact receptors cannot be excluded, which may limit discrimination between viable and nonviable bacteria.

SEM images showed both normal rod-shaped *E. coli* cells and a subpopulation with visibly altered morphology within the same preparation (Figure 9), suggesting that the effect is not solely attributable to a uniform fixation artifact. Instead, the coexistence of both morphotypes is consistent with a state-dependent susceptibility of the cell envelope to preparation- and vacuum-related stresses.

**Figure 9:**
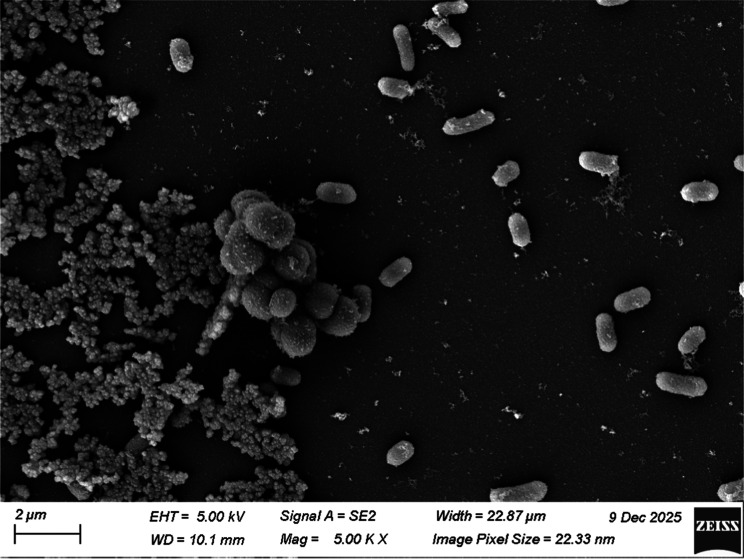
SEM images showing associations between phage@CMD particles and *E. coli* cells after magnetic separation. A subpopulation of cells displays altered morphology consistent with envelope perturbation.

Chemical fixation and dehydration required for SEM can differentially affect cells with compromised envelope integrity, leading to swelling or shape changes during sample processing. Such fixation-associated swelling and partial lysis phenomena have been reported for *E. coli* in bacteriophage contexts when cell–wall–degrading activity persists during fixation.^49^

In our system, the observed swelling/rounding is therefore plausibly linked to local envelope destabilization triggered by phage–host interactions, which would render a fraction of cells more sensitive to dehydration and high-vacuum SEM conditions. This interpretation is consistent with established phage lysis mechanisms in which holin-mediated membrane permeabilization enables endolysin access to the peptidoglycan, weakening the cell wall prior to rupture. Reports of T4 infection further describe rod-to-more-spherical transitions in *E. coli* preceding lysis, supporting the plausibility of lysis-like morphological changes.^50^

Accordingly, we interpret the SEM morphology as qualitative support for bacteria–particle interactions and envelope perturbation, while noting that SEM sample preparation and vacuum exposure can amplify morphological differences between intact and envelope-compromised cells.

The microscopy observations are consistent with the CFU and PCR results and provide direct structural evidence for bacteria–particle interactions mediated by immobilized phages.

Overall, these results demonstrate that phage@CMD particles enable selective magnetic enrichment of *E. coli*, with capture efficiencies determined by the functional accessibility of immobilized phages rather than total loading density.

## Conclusion

In this work, CMD-coated magnetic nanoparticle clusters functionalized with lytic T4 bacteriophages were developed and evaluated as a magnetic preconcentration system for selective *E. coli* capture prior to PCR analysis. Comprehensive physicochemical characterization confirmed multicore particle morphology, superparamagnetic behavior, and successful surface functionalization enabling covalent phage immobilization.

Semiquantitative assessment of phage loading demonstrated that high immobilization densities can be achieved using EDC/Sulfo-NHS coupling; however, functional capture efficiencies remained moderate (≈20–40%). This finding highlights that immobilization-derived loading does not directly translate into proportional biological performance, likely due to nonoriented attachment, steric constraints, and partial loss of functional accessibility of surface-bound phages.

Magnetic separation experiments combined with PCR analysis demonstrated that phage-mediated enrichment improves practical PCR detectability by increasing the amount of bacterial DNA available for amplification. SEM observations further supported bacteria–particle interactions and suggested local envelope perturbation consistent with phage-mediated effects.

Overall, this study provides an experimental framework to investigate the relationship between phage immobilization, surface loading, and functional bacterial capture. The results emphasize practical design constraints of nonsite-specific covalent coupling and provide guidance for future optimization strategies aimed at improving functional accessibility and capture performance in phage-based magnetic preconcentration systems.

## Supplementary Material



## Data Availability

The raw data supporting the findings of this study will be made available in the Zenodo repository. The raw data supporting the findings of this study are available in the Zenodo repository at https://doi.org/10.5281/zenodo.20530941.

## Notes

Table S1: Dilution scheme of phage suspension for double-layer agar (DLA) assay. Figure S1: Time-course of bacteriophage release during a one-step growth experiment at MOI 0.001. Figure S2: Normalized phage release kinetics at MOIs 0.01 and 0.001. Figure S3: Binding kinetics of bacteriophage T4 to *E. coli*. Figure S4: TEM image of free T4 phages prior to immobilization. Figure S5: Vibrating sample magnetometry (VSM) of CMD-coated particles. Figure S6: FT-IR spectrum of CMD-coated particles with characteristic band assignments. Figure S7: DLS size distributions of free phages, CMD particles, and phage@CMD conjugates. Figure S8: TEM images of phage@CMD particles. Figure S9: FT-IR spectra of CMD particles after EDC/Sulfo-NHS activation. Figure S10: FT-IR control spectra without EDC/Sulfo-NHS activation. Figure S11: Agarose gel electrophoresis for *L. plantarum* selectivity assessment. Figure S12: Double-layer agar (DLA) assay images for phage concentration determination (PDF)
