# Supplementary material for: Phage-Functionalized Magnetic Separation for Rapid and Selective Detection of Escherichia coli
Source: ACS Omega. 2026 Jun 15;11(25):37605–18. doi: 10.1021/acsomega.6c02406 (PMC13325175; doi:10.1021/acsomega.6c02406)
Supplement: Supplementary file 1 [file ao6c02406_si_001.pdf]

Supporting Information

Phage-functionalized magnetic separation for rapid and selective detection of *Escherichia coli*

Marco Eigenfeld[1,2,*]; Benjamin Schneider[1]; Dagmar Kolb[2,3,4]; Ana Lisac[5]; Ales Podgornik[5]; Sebastian P. Schwaminger[1,2,*]

[1]Medical University of Graz, Otto Loewi Research Center, Division of Medicinal Chemistry, NanoLab; Neue Stiftingtalstraße 6, 8010 Graz, Austria
[2]BioTechMed-Graz, Mozartgasse 12/II, 8010 Graz, Austria
[3]Medical University of Graz, Core Facility Ultrastructural Analysis, Neue Stiftingtalstraße 6, 8010 Graz, Austria
[4]Medical University of Graz, Gottfried Schatz Research Center, Division of Cell Biology, Histology and Embryology, Neue Stiftingtalstraße 6, 8010 Graz, Austria
[5]Faculty of Chemistry and Chemical Technology, University of Ljubljana, Večna pot, 113, Ljubljana, Slovenia

*Corresponding authors: marco.eigenfeld@medunigraz.at; sebastian.schwaminger@medunigraz.at

Supporting Information

Table S1: Dilution scheme of phage suspension for DLA

Tube	*Dilution Factor*	*Volume Transferred*	*Diluent Volume*	*Total Volume*	*Final Concentration*
Stock	10^0	—	—	—	1.07×10^{10}
1	10^{-1}	100 µl stock	900 µl	1 ml	1.07×10^9
2	10^{-2}	100 µl tube 1	900 µl	1 ml	1.07×10^8
3	10^{-3}	100 µl tube 2	900 µl	1 ml	1.07×10^7
4	10^{-4}	100 µl tube 3	900 µl	1 ml	1.07×10^6
5	10^{-5}	100 µl tube 4	900 µl	1 ml	1.07×10^5

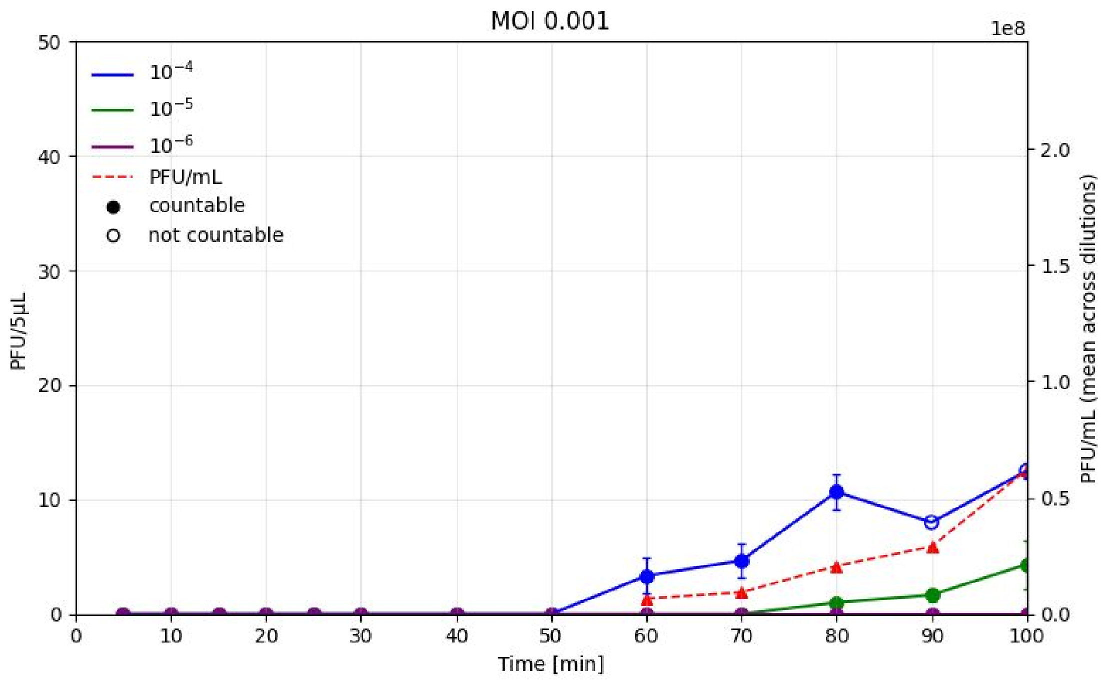

Figure S1: Time-course of bacteriophage release during a one-step growth experiment at MOI 0.001. Samples were taken at indicated time points post-infection and plated as 5 µL droplets on double-layer agar (DLA) at three serial dilutions (10^{-4}, 10^{-5}, and 10^{-6}); MOI=0.001

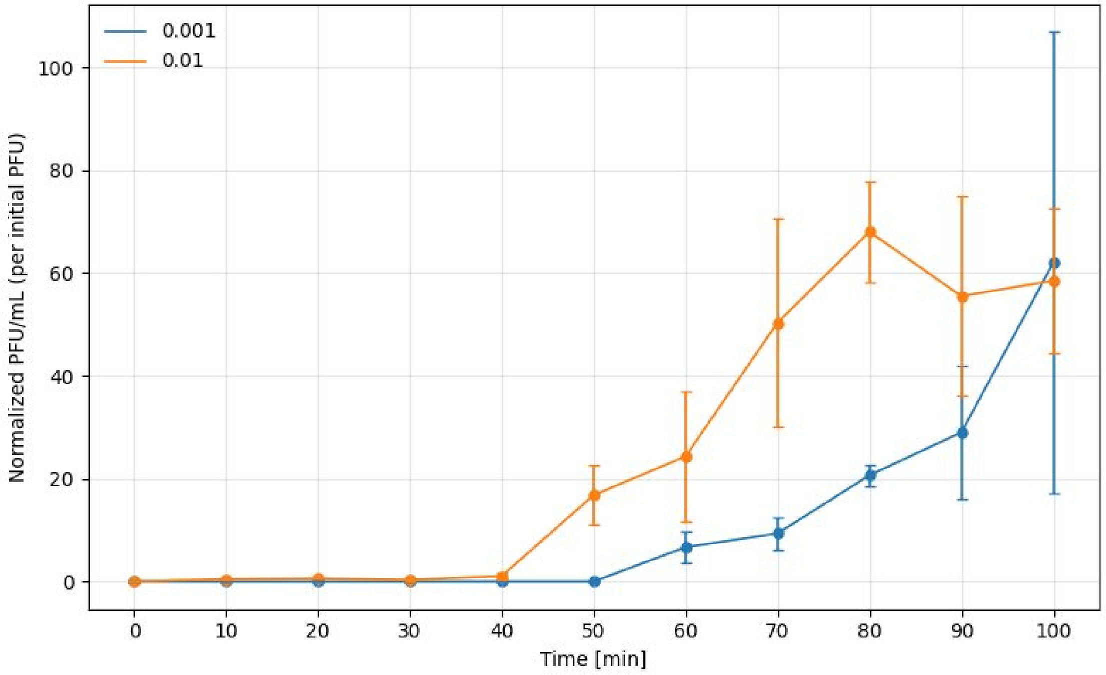

Figure S2: Normalized time-course of bacteriophage release during a one-step growth experiment at MOI 0.01 and 0.001.

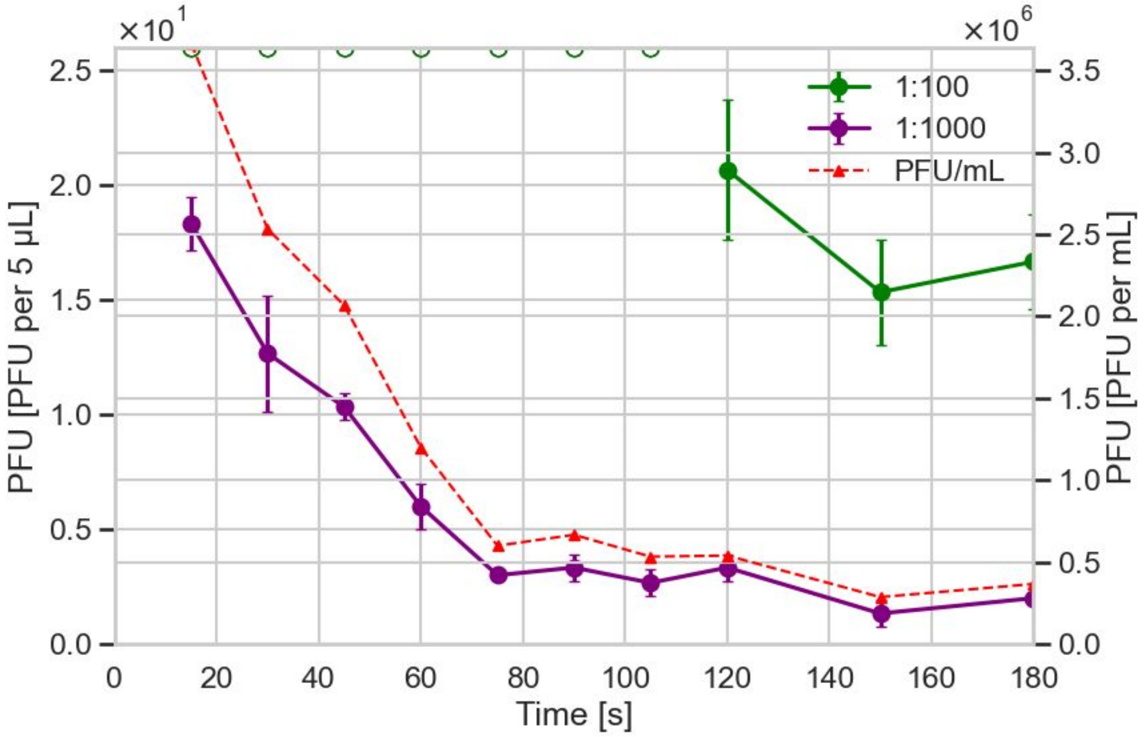

Figure S3: Binding kinetics of the bacteriophage to *E. coli*.

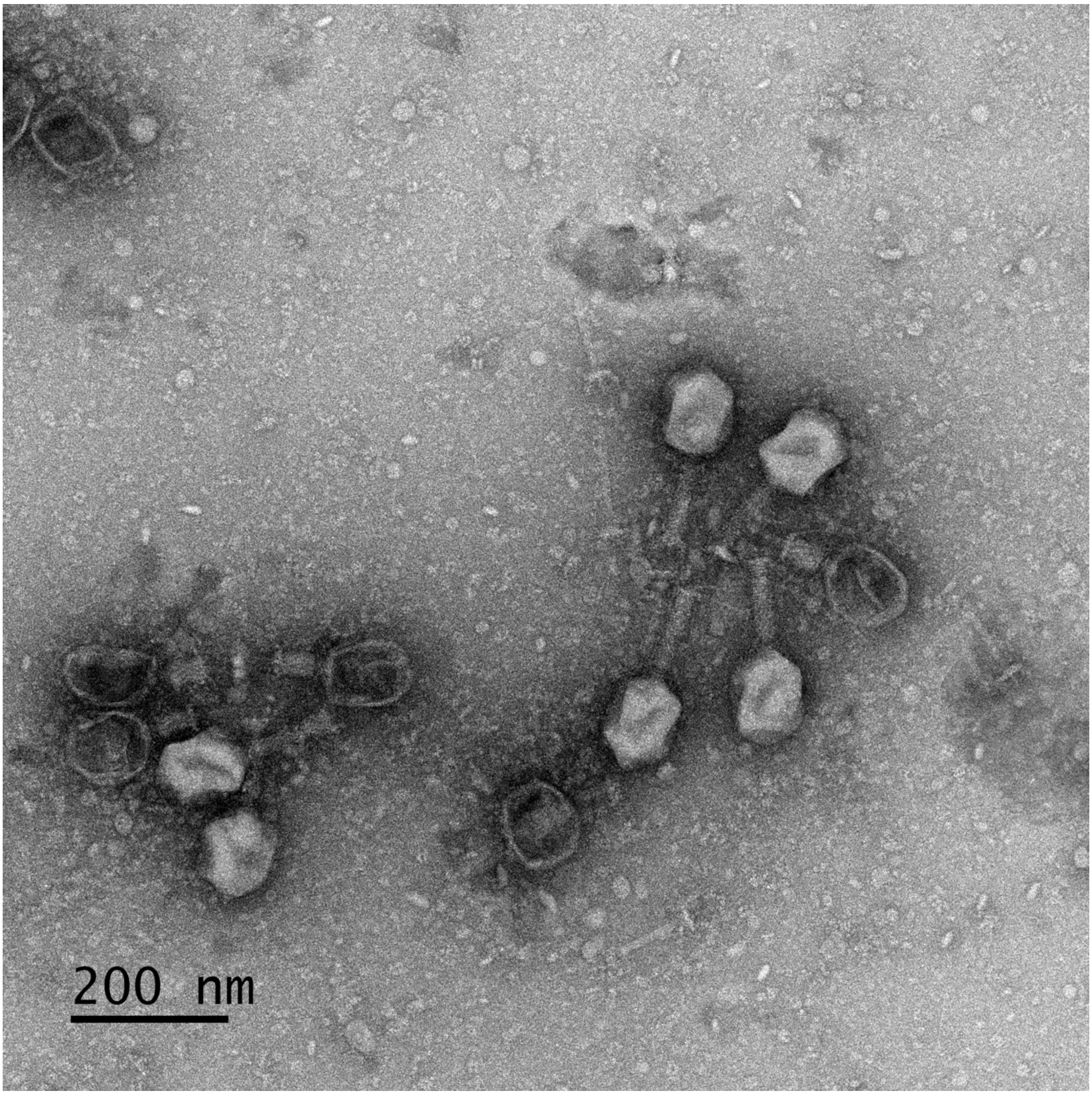

Figure S4: TEM image of phage only sample

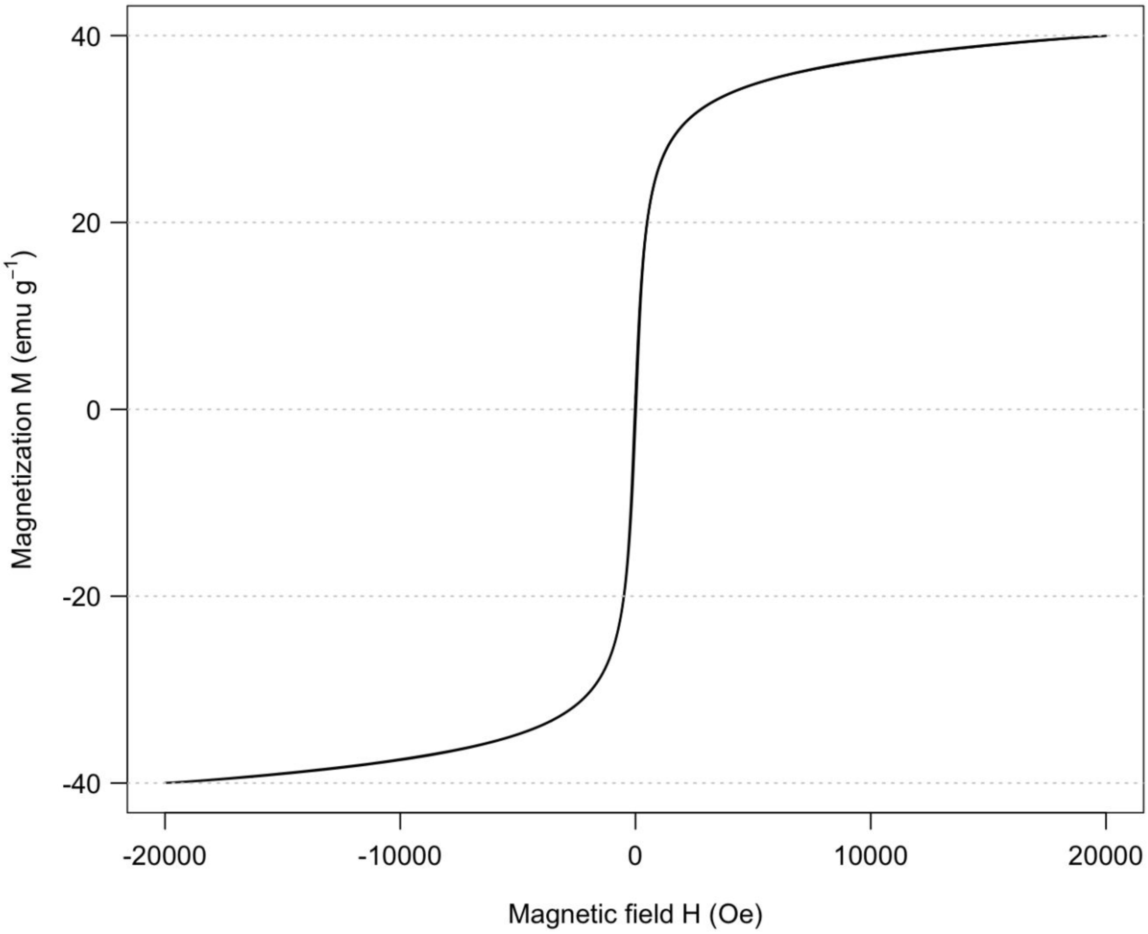

Figure S5: VSM measurement of CMD coated particles

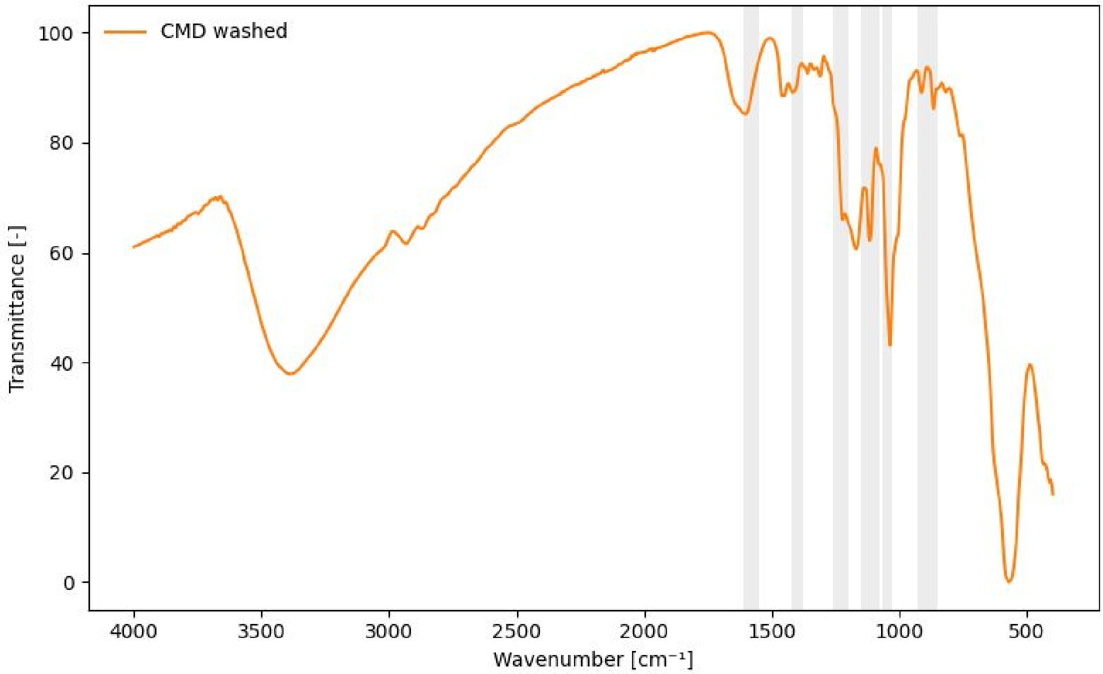

Figure S6: FT-IR spectrum of CMD particles; The gray-shaded regions indicate characteristic vibrational bands of CMD: 1550–1610 cm^{-1} (COO^{-} asymmetric stretch), 1420–1380 cm^{-1} (COO^{-} symmetric stretch), 1260–1200 cm^{-1} (C–O–H stretching), 1150–1080 cm^{-1} (C–O–C glycosidic vibrations), 1070–1030 cm^{-1} (C–O stretching), and 930–850 cm^{-1} pyranose ring vibrations).

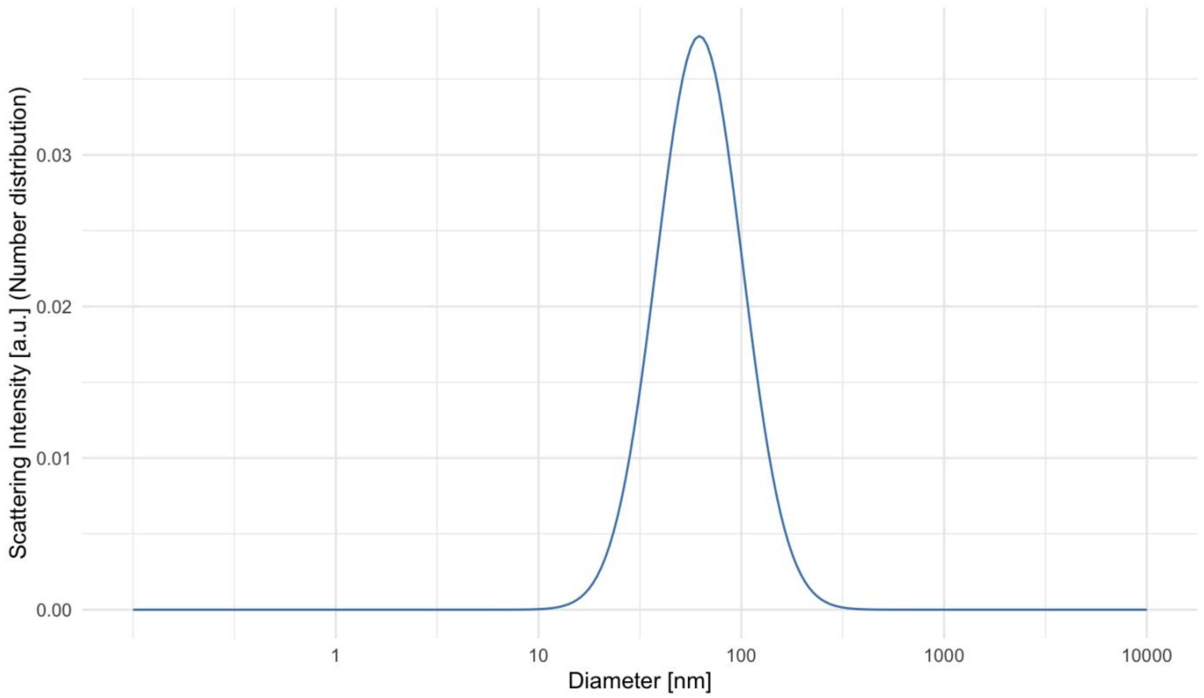

Figure S7: Number distribution of CMD coated BIONs

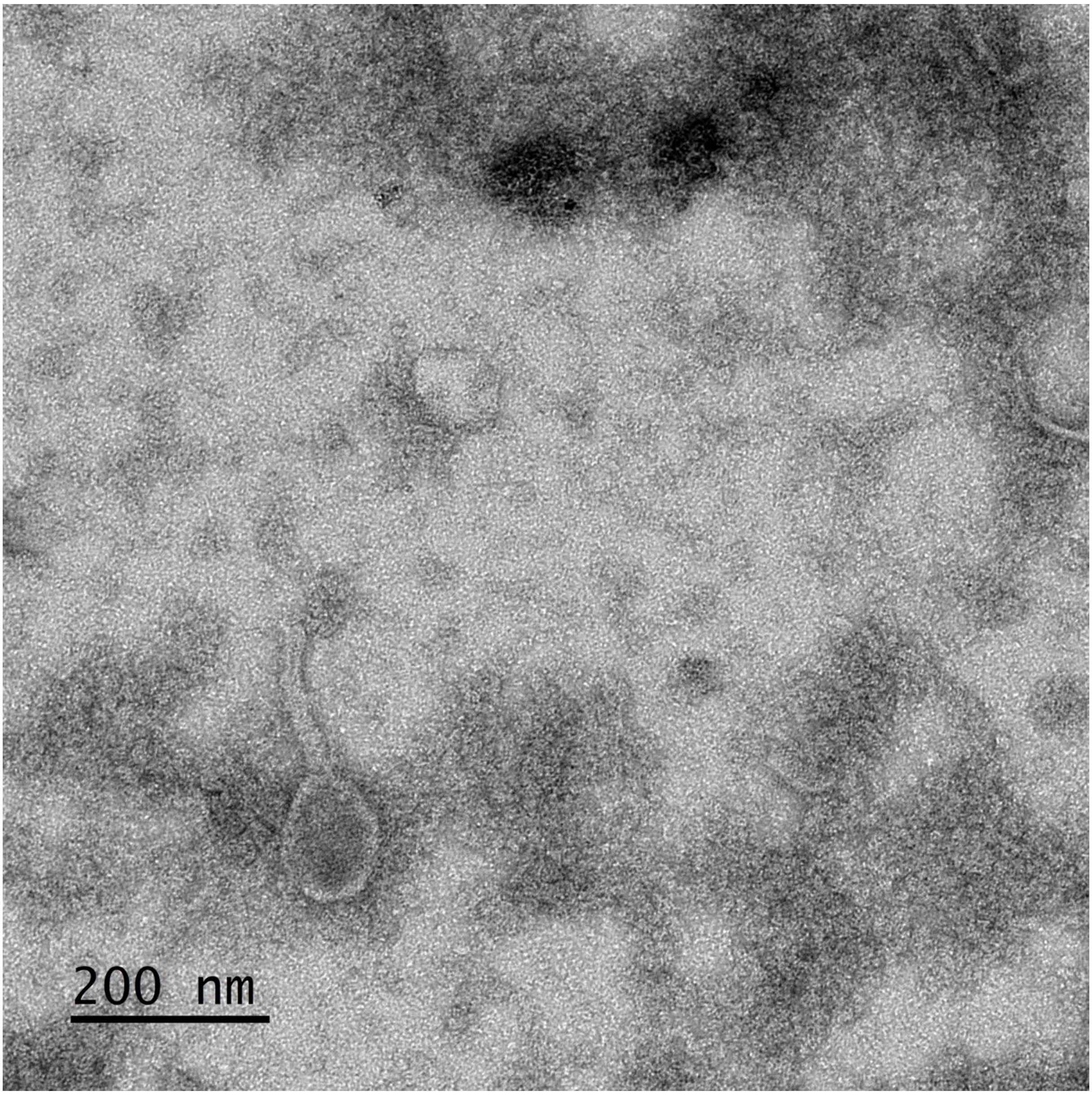

Figure S8: TEM of phage@CMD particles

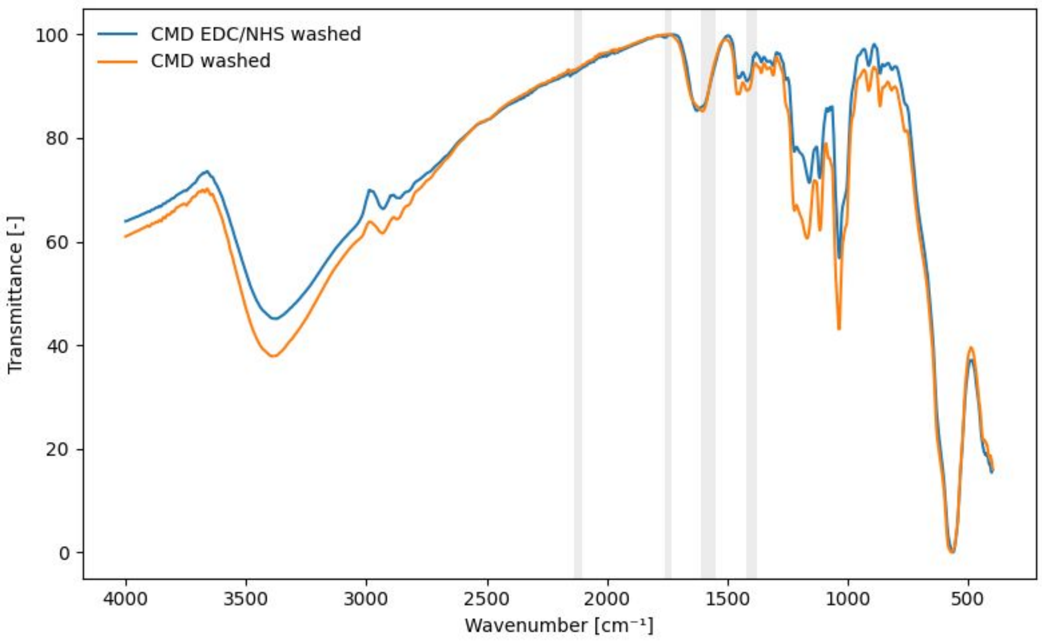

Figure S9: FT-IR spectra of washed CMD particles and CMD particles modified with EDC/NHS after three times wash with activation buffer. the gray-shaded regions indicate characteristic vibrational bands of EDC and NHS: 1550–1610 cm^{-1} (COO^- asymmetric stretch), 1420–1380 cm^{-1} (COO^- symmetric stretch), 1735–1760 cm^{-1} (NHS-Ester-C=O), 2105–2135 cm^{-1} (-N=C=N (EDC)

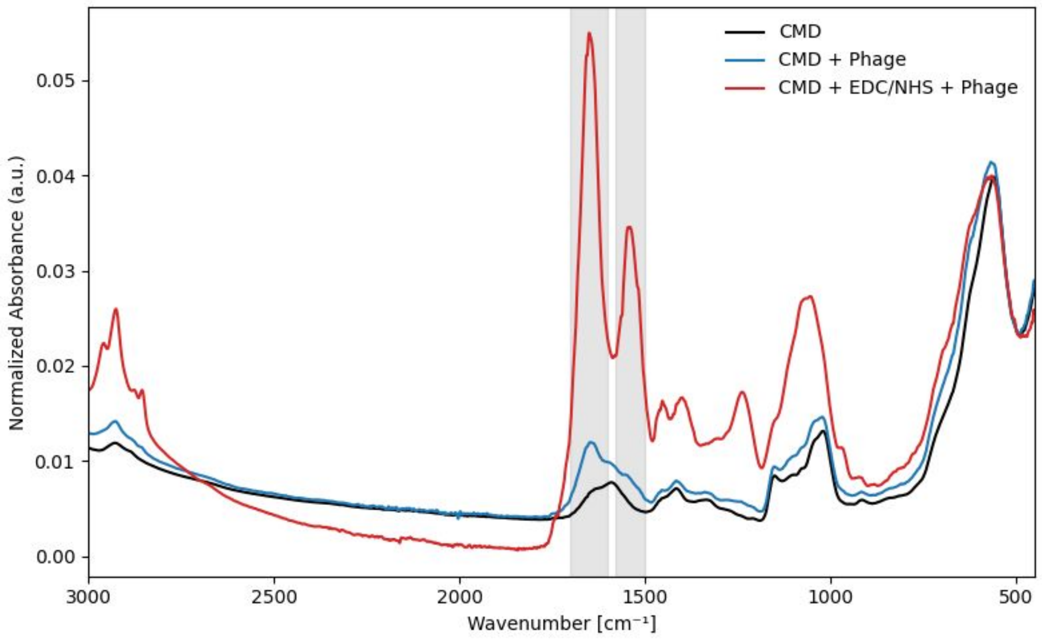

Figure S10: FT-IR spectra of CMD particles, CMD particles incubated with phages (without EDC/Sulfo-NHS), and CMD particles functionalized with EDC/Sulfo-NHS and phages after two washing steps.

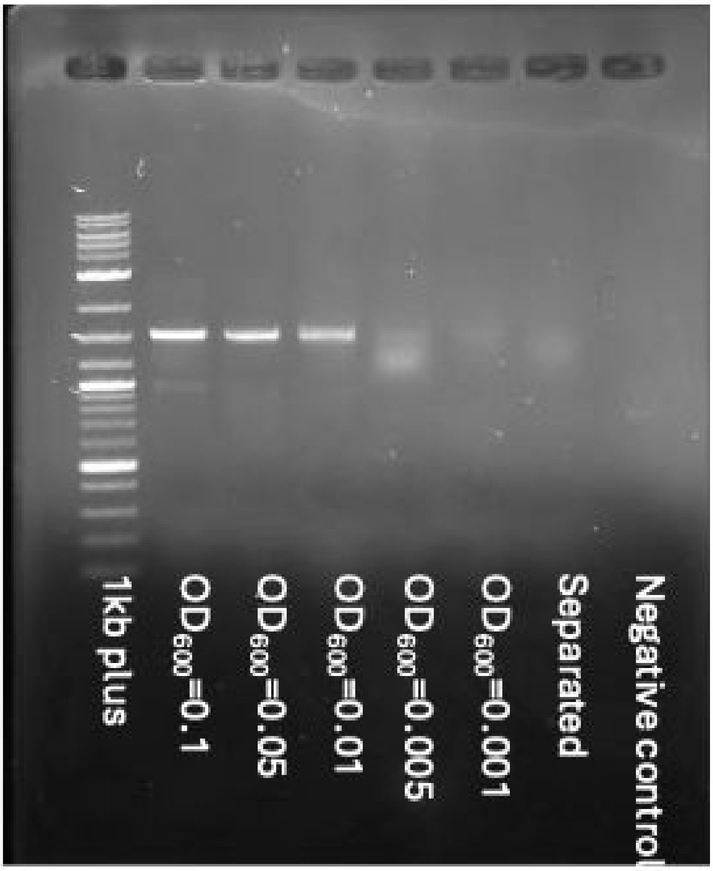

Figure S11: Agarose gel electrophoresis of PCR products obtained from *L. plantarum* cultures at various optical densities (OD_{600}) and from the separated *L. plantarum* fraction after incubation with phage-functionalized CMD particles.

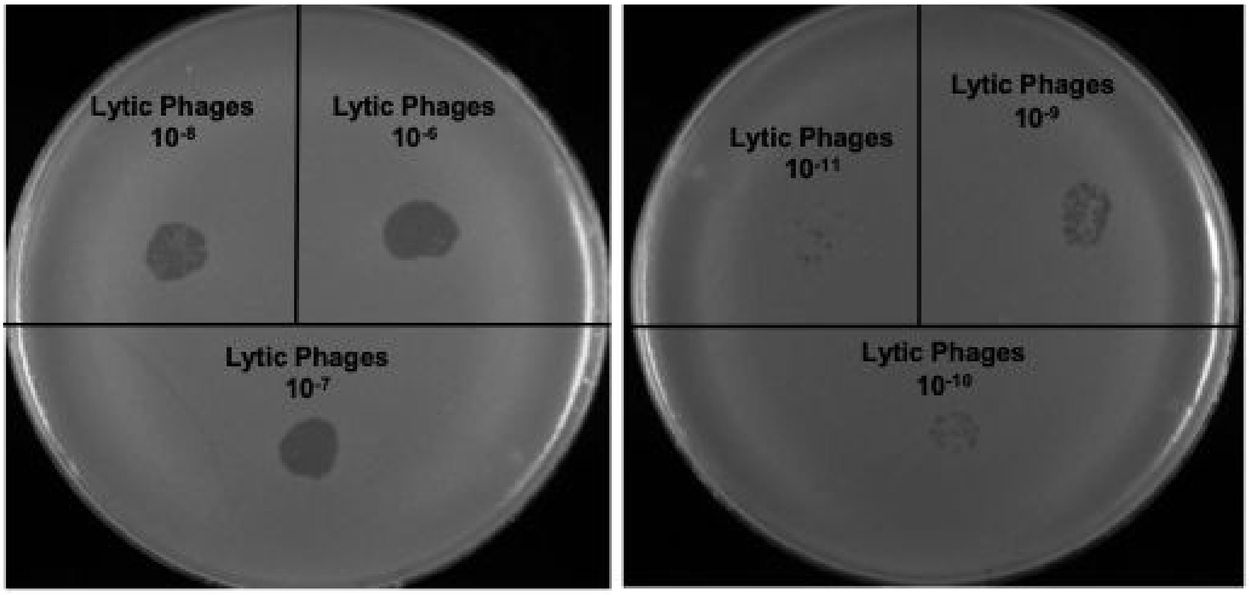

Figure S12: Double-layer agar (DLA) assay of lytic T4 phages for concentration determination using a 5-µL drop.
